# Variation in the effectiveness of insecticide treated nets against malaria and outdoor biting by vectors in Kilifi, Kenya

**DOI:** 10.12688/wellcomeopenres.11073.4

**Published:** 2018-12-03

**Authors:** Alice Kamau, Joseph M. Mwangangi, Martin K. Rono, Polycarp Mogeni, Irene Omedo, Janet Midega, J. Anthony G. Scott, Philip Bejon

**Affiliations:** 1 KEMRI-Wellcome Trust Research Programme, Centre for Geographic Medicine Research-Coast, Kilifi, Kenya; 2 Integrated Vector and Disease Management Cluster, International Centre of Insect Physiology and Ecology, Nairobi, Kenya; 3 Pwani University Bioscience Research Centre, Pwani University, Kilifi, Kenya; 4 Centre for Genomics and Global Health, Wellcome Trust Centre for Human Genetics, University of Oxford, Oxford, OX3 7BN, UK; 5 Department of Infectious Disease Epidemiology, London School of Hygiene & Tropical Medicine, London, WC1E 7HT, UK; 6 Centre for Tropical Medicine and Global Health, Nuffield Department of Clinical Medicine, University of Oxford, Oxford, OX3 7FZ, UK

**Keywords:** ITNs, outdoor, Anopheles mosquito, effectiveness, Kilifi, Kenya, KHDSS

## Abstract

**Background**: Insecticide treated nets (ITNs) protect humans against bites from the *Anopheles *mosquito vectors that transmit malaria, thereby reducing malaria morbidity and mortality. It has been noted that ITN use leads to a switch from indoor to outdoor feeding among these vectors. It might be expected that outdoor feeding would undermine the effectiveness of ITNs that target indoors vectors, but data are limited.

**Methods**: We linked homestead level geospatial data to clinical surveillance data at a primary healthcare facility in Kilifi County in order to map geographical heterogeneity in ITN effectiveness and observed vector feeding behaviour using landing catches and CDC light traps in six selected areas of varying ITN effectiveness. We quantified the interaction between mosquitoes and humans to evaluate whether outdoor vector biting is a potential explanation for the variation in ITN effectiveness.

**Results**: We observed 37% and 46% visits associated with positive malaria slides among ITN users and non-ITN-users, respectively; ITN use was associated with 32% protection from malaria (crude OR = 0.68, 95% CI: 0.64, 0.73). We obtained modification of ITN effectiveness by geographical area (p=0.016), and identified 6 hotspots using the spatial scan statistic. Majority of mosquitoes were caught outdoor (60%) and were of the *An. funestus* group (75%). The overall propensity to feed at times when most people were asleep was high; the vast majority of the *Anopheles* mosquitoes were caught at times when most people are indoors asleep. Estimates for the proportion of human-mosquito contact between the first and last hour when most humans were asleep was consistently high across all locations, ranging from 0.83 to 1.00.

**Conclusion**: Our data do not provide evidence of an epidemiological association between microgeographical variations in ITN effectiveness and variations in the microgeographical distribution of outdoor biting.

## Introduction

Despite the recent scale-up effort to achieve control, malaria continues to cause morbidity and mortality, especially in sub-Saharan Africa. There are uncertainties in global estimates ^1– 3^; however in 2015, the World Health Organization estimated global deaths due to malaria to be 438,000 (range: 236,000–635,000) and the burden of febrile illness at 214 million cases (range: 149–303 million) ^4^. Estimates from model-based predictions suggest that approximately 1.4 billion of the global population live at risk of stable malaria and ~1.1 billion at risk of unstable malaria ^5^.

The frontline tools for malaria control in sub-Saharan Africa, insecticide treated nets (ITNs) and indoor residual spray, are most effective if baseline transmission occurs indoors ^6^. The major vectors of human malaria mostly feed indoors, and transmission can therefore be substantially reduced by these tools ^6^. The proportion of the at risk population who have access to ITNs was modeled to have increased from 4% to 67% between 2004 and 2015 ^7^. ITNs operate in three ways: deterrence, excito-repellence and killing, thereby reducing the density, feeding frequency, feeding success, and survival of *Anopheles* mosquito vectors ^6^. By reducing vector densities and vector survival, ITNs not only directly protect the individual ITN user, but also reduce the overall transmission intensity and protect the whole community when a particular threshold of bed net coverage is reached ^8– 10^. The evidence base supports ITN use over a range of transmission intensities ^11^ and protective efficacy has been demonstrated against infection, clinical disease and mortality ^12– 16^. However, residual malaria transmission is well described even after optimal ITN use, which could be associated with outdoor biting behaviour of the mosquito vector that allows them to evade fatal contact with these frontline tools of intervention ^17, 18^. The most obvious behavioural change is the mosquito vector exhibiting exophagic tendencies –i.e. the vector feeds outdoors.

Among malaria vectors in Africa, the two principal taxa are: *Anopheles gambiae sensu lato* (s.l.) and *Anopheles funestus* group. Both species complexes feed primarily indoors; however, both have exhibited outdoor biting or feeding in the early part of the evening in some areas where ITNs have been deployed ^6, 19– 22^. This behavioral change might have resulted from one of three processes: (i) selection, either for species that more readily engages in outdoor feeding, for instance in favour of *An. arabiensis* rather than *An. gambiae sensu strictu* (s.s.); (ii) by selecting for evolutionary change within a species; or (iii) a response to inability to feed during the night in the absence of genetic variation ^23, 24^. In Western Kenya and South-eastern Tanzania there have been reports of a reduction in indoor feeding by *An. gambiae sensu stricto* ( *s.s*.) and an increase in the relative abundance of *An. arabiensis.* The latter has a broader range of feeding times and biting behavior, including: feeding at dusk or dawn on humans outdoors; readily feeding on animals when available; or repeatedly foraging inside houses until an unprotected non-ITN user is found ^8, 17, 23, 25, 26^. In southern Tanzania, where ITNs have been used for several years, the mosquitoes are biting more frequently during the hours of the early evening and early morning when people are more likely to be awake and vulnerable outside of their nets ^6, 27^. The potential for ITNs to result in species switches was appreciated in earlier controlled trials ^23, 26, 28^, and is now reported more widely as ITN use is scaled up in Western Kenya and on the East African coast ^23, 26^.

In Kilifi, Kenya, a switch in the most common vector, from *An. gambiae sensu stricto (s.s.)* to *An. arabiensis*, occurred during the period of ITN scale-up ^22^. The increased ability of *An. arabiensis* to feed outdoors might be expected to result in a decrease in ITN effectiveness. However, there is little data to support this contention, and some data and models that are available suggest that ITNs continue to be effective despite outdoor feeding ^29, 30^. The objectives of this study were (i) to examine whether there has been a shift in vector biting patterns and/or vector behaviour, during the period of intense ITN use along the Kenyan coast; (ii) to test for geographical heterogeneity in ITN effectiveness within the surveillance area of a primary healthcare facility in Kilifi County; and (iii) to assess whether outdoor vector biting is a potential explanation for the variation in ITN effectiveness.

## Methods

### Study area

**Figure 1.**
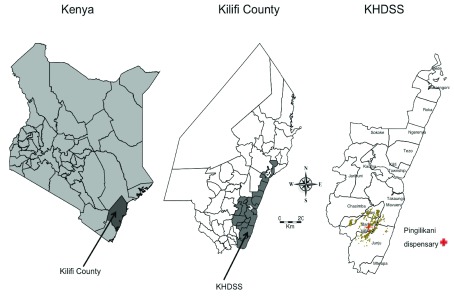
Situation of Kilifi County in Kenya and the map of Kilifi County showing the boundaries of the KHDSS. The map of KHDSS shows the locations and the situation of homesteads and Pingilikani dispensary where the study was conducted. The brown plotted point on the KHDSS map represents homesteads.

The clinical surveillance study was conducted between January 2009 and December 2016 within a 6km radius of Pingilikani dispensary in Kilifi County on the Kenyan Coast ( Figure 1): within the Kilifi Health and Demographic Surveillance System (KHDSS). All children under 13 years presenting for medical assessment to Pingilikani dispensary (except those with trauma as their only concern) were assessed by research staff and had finger-prick blood samples examined for malaria parasites. Thick and thin blood smears were stained with 10% Giemsa and examined at 1000X magnification for asexual *Plasmodium falciparum* parasites. Before slides could be considered negative, 100 fields were examined. Children with malaria positive slides were treated with co-artemether.

**Figure 2.**
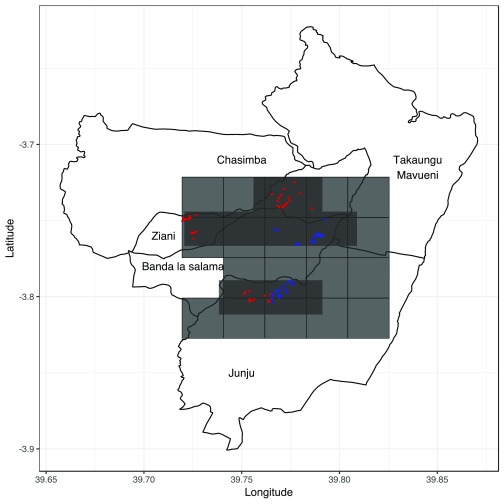
Map of the 2.5×2.5 km geographical areas (grids in light gray), the geographical areas where mosquito sampling was conducted (grids in dark gray) and the homesteads where mosquito sampling was done. Each plotted point represents an individual homestead, where color shading indicates ITN effectiveness, with red shading indicating low effectiveness and blue shading indicating high effectiveness.

Transmission of malaria peaks after the long rains from April to June and the short rains from October to November each year, although transmission has been declining ^31– 34^. The surveillance area was divided into 2.5×2.5 km regular polygons resulting in 21 geographical areas ( Figure 2). As part of KHDSS, four-monthly enumeration rounds were conducted to identify births, deaths and migration events. Each inhabitant was described by their family relationships and their homestead of residence, with geospatial coordinates, and assigned a unique personal identifier ^35^. These details were used to link children visiting Pingilikani dispensary to geospatial coordinates for the homestead of residence. Data on ITN use was collected once yearly during cross-sectional surveys integrated into the regular KHDSS enumeration since 2008. Questionnaires were used to collect household data on ITN ownership and use on the night prior to enumeration ^36^. Six geographical areas were selected for mosquito sampling out of 21 areas for which clinical effectiveness estimates were determined ( Figure 2). The basis of selecting the six areas was (i) geographical areas with >60 homesteads available for randomization; (ii) areas representing varying ITN effectiveness.

### Mosquito sampling

Indoor and outdoor biting profiles of *An. gambiae s.l.* and the *An. funestus* group were estimated using human landing catches (HLC) and CDC-light traps (CDC-LT) by visiting randomly selected houses (random selection done by stratified sampling) between July and August 2016. For both indoor and outdoor mosquito collection, HLC was conducted by two pairs of trained male volunteers (one pair was located indoors and the other pair outdoors, but at the same homestead), who sat with their legs exposed and caught mosquitoes that attempted to bite them using an aspirator. HLC was conducted between 18:00hours and 06:00hours for 45 minutes each hour, allowing 15 minutes break for rest. The catches for each hourly interval were stored in separate collection cups. CDC-light traps were also set indoor and outdoor between 18:00hours and 06:00hours. The HLC and the CDC-LT collections took place in different houses. In each geographical area, sampling was conducted for at least 3 days in at least 16 houses; 8 houses for HLC and 8 houses for CDC-LT. In total, 26 days of sampling were conducted across 115 houses in the six selected geographical areas within the surveillance area.

### Mosquito processing

The mosquito samples were morphologically separated for sex and identified for species ^6, 27^. The female *Anopheles* mosquitoes were tested for *falciparum* infection using a sandwich circumsporozoite protein (CSP) enzyme linked immunosorbent assay (ELISA) ^37^ (anti-CSP capture: Pf2A10-28 and conjugate : Pf2A10-CDC antibodies; KPL, Gaithersburg, MD, USA). Individual mosquitoes were stored at -20°C in micro-centrifuge tubes containing a small amount of desiccant (silica gel) separated from the mosquito by a thin layer of cotton prior to ELISA and molecular analysis for sibling species by polymerase chain reaction ^38, 39^.

### Human behaviour

To determine the human-mosquito contact, we administered questionnaires to 304 randomly selected households in the six geographical areas between September and October 2016. We asked the household head time when each household member went to sleep and the time they woke up. Data on human behaviour was used to make adjustments to the indoor and outdoor biting rate.

## Statistical analysis

### Geographical variations in ITN effectiveness

Statistical analyses were performed using STATA v13.1 (StataCorp, College Station, TX, USA). To assess for geographical heterogeneity, we used the logistic regression model to analyze data on over 20,000 visits from children attending Pingilikani dispensary. The outcome of interest was presence of malaria by microscopy on presentation to the dispensary. The potential risk factors included: ITN use, age of the child, year of presentation to the dispensary, season (the wet season comprised of April, May, June, October and November) and the geographical area, as defined by the 2.5×2.5 km regular polygons. We assessed whether the effect of ITN use on malaria was altered by geographical area by including an interaction term between geographical area and ITN use. We also assessed whether the effect of ITN use was altered by the age of the child and whether geographical areas altered the effect of age. To assess the nonlinear effect of age in the regression models, multiple fractional polynomial transformation was used ^40^. A list of fractional polynomial (FP) powers (–2, –1, –0.5, 0, 0.5, 1, 2, 3) were investigated for inclusion in the model using an algorithm that combines a backward elimination procedure with a search for an FP function that best predicts the outcome variable as previously described ^41^. Given that the hospital malaria episodes were clustered within patients, we allowed for clustering by using a logistic regression model with robust standard errors ^42^. The robust standard errors were used to account for the clustering effect in the estimation of the standard errors. The ratio of malaria in the non-ITN users to that in the ITN users was expressed as an odds ratio (OR) as determined by logistic regression. ITN effectiveness was calculated as (1 – OR) × 100. Model fit was assessed by examining residuals against covariates. ITN effectiveness was also computed for each individual homestead aggregated at a 2.5 km smoothing and without smoothing. Spearman’s rank correlation was used to assess the association between ITN effectiveness and prevalence of malaria. SaTScan software (version 9.4; https://www.satscan.org/), a spatial scan statistic developed by Kulldorf ^43^, was used to detect potential spatial variations of ITN effectiveness (without smoothing) by identifying statistically significant geographical clustering of ITN effectiveness using the normal model. The space-time parameter of the spatial scan statistic places a cylindrical window on the coordinates grid for the locations studied and moves the center of the cylinder base over the grid so that the sets of geographic units covered by the window are constantly changing. Whenever the cylindrical window includes a new event, SaTScan calculates a likelihood function to test for elevated risk within the cylinder as compared with outside the cylinder. The observed test statistic is obtained by calculating the likelihood ratio maximized over the collection of zones in the alternative hypothesis. The p value for the detection of clusters is calculated by using the Monte Carlo hypothesis testing (where a number of random replications of the dataset under the appropriate null hypothesis are generated, their test statistics computed and then compared with the observed test statistic to obtain the p-value). The null hypothesis is that the risk of malaria inside and outside the scanning window is the same ^43^.

### Vector abundance

In order to compare counts of female *Anopheles* captured, we determined the relative proportion of each mosquito species in each geographical area and ITN effectiveness levels (ITN effectiveness was divided into 2 levels based on the estimates obtained from the logistic regression above –i.e. high and low ITN effectiveness). Three areas (6, 15 & 16) with high ITN effectiveness and three areas (5, 19 & 20) with low ITN effectiveness were selected based on the findings of the scan statistic. We compared the proportion of vectors biting outdoors and those caught outside of sleeping hours in each geographical area. We estimated the confidence intervals of these proportions using the binomial distributions, and tested for an association between biting preference and ITN effectiveness (at the level of geographical area) using the Spearman’s rank correlation.

### Human behaviour

Questionnaire data about the time household members went to sleep and at what time they woke up were combined with human landing catches measurements of hourly rates for indoor and outdoor biting. The proportion of people indoor and outdoor at each hour of the night was calculated. We estimated the proportion of human exposure to mosquito bites occurring indoors (π _s_) by taking into consideration the movement pattern of people using the following method ^44^: by weighting the mean indoor and outdoor biting rates throughout the night by the proportion of humans reporting to have gone to sleep at each hour of the night, as an indicator of the upper limit of personal protection that indoor vector control measures can provide, as follows;

$$\pi_{s}=\sum_{t=1}^{12}\left(B_{i,t}S_{t}\right)/\sum_{t=1}^{12}\left(B_{i,t}S_{t}+B_{o,t}(1-S_{t})\right)$$

Where:

π _s_ = an estimate of human exposure to bites which occurs when residents are both indoors and sleeping

S _*t*_ = the proportion of humans indoors reporting to have gone to sleep at each hour of the night (t)

B _*i, t*_ = mean indoor biting rate at each hour of the night (t)

_B *o, t*_ = mean outdoor biting rates at each hour of the night (t)

(1-S _*t*_) = proportion of humans not yet asleep at each hour of the night

## Results

### Geographical variations in ITN effectiveness

Between 2009 and 2016, there were 29,187 visits to Pingilikani dispensary made by 5,800 children aged between 3 months to 12 years ( Table 1). Of these visits, 11,505 (39.4%) were classified as episodes of malaria, with a median number of 9 (IQR: 5, 15) episodes per child during this time period. The number of children, cases of malaria and ITN use in the 21 geographical areas examined is summarized in detail in Table 1. ITN use was consistently >50% in all geographical areas and the prevalence of ITN use in non-malaria cases was 74.2% (95% CI: 73.5, 74.8).

**Table 1.** Description of insecticide treated net (ITN) use and cases of malaria in the 2.5×2.5 km geographical areas.

Areas	Children	Visits	Malaria visits- ITN user n (%)	Non-malaria visits ITN user n (%)	Malaria visit non-user n (%)	Non-malaria visits non-user n (%)	Malaria prevalence (%)	ITN use (%)
1	13	15	5 (0.07)	6 (0.05)	3 (0.08)	1 (0.02)	53.3	73.3
2	17	25	11 (0.14)	12 (0.09)	0(0)	2 (0.04)	44	92
3	4	6	1 (0.01)	5 (0.04)	0(0)	0 (0)	16.7	100
4	5	10	5 (0.07)	3 (0.02)	1 (0.03)	1 (0.02)	60	80
5	275	1232	484 (6.35)	481 (3.67)	122 (3.14)	150 (3.13)	49.2	78.3
6	690	4335	1264 (16.59)	1909 (14.55)	587 (15.10)	612 (12.78)	42.7	73.2
7	6	6	0(0)	5 (0.04)	0(0)	1 (0.02)	0	83.3
8	173	348	62 (0.81)	148 (1.13)	52 (1.34)	88 (1.84)	32.8	60.3
9	42	201	48 (0.63)	78 (0.59)	32 (0.82)	54 (1.13)	39.8	62.7
10	1343	9639	2910 (38.20)	4467 (34.05)	1055 (27.14)	1277 (26.67)	41.1	76.5
11	308	1284	453 (5.95)	502 (3.83)	205 (5.27)	130 (2.72)	51.3	74.4
12	19	40	6 (0.08)	18 (0.14)	4 (0.10)	12 (0.25)	25	60
13	497	1109	148 (1.94)	617 (4.70)	99 (2.55)	245 (5.12)	22.3	68.9
14	212	1136	219 (2.87)	384 (2.93)	256 (6.59)	303 (6.33)	41.8	53.1
15	605	3704	682 (8.95)	1672 (12.74)	602 (15.49)	770 (16.08)	34.7	63.6
16	567	2881	623 (8.18)	1125 (8.57)	551 (14.18)	602 (12.57)	40.8	60.7
17	29	40	5 (0.07)	26 (0.20)	3 (0.08)	6 (0.13)	20	77.5
18	49	206	67 (0.88)	80 (0.61)	32 (0.82)	29 (0.61)	48.1	71.4
19	520	1911	418 (5.49)	1047 (7.98)	160 (4.12)	295 (6.16)	30.3	76.7
20	423	1055	206 (2.70)	535 (4.08)	122 (3.14)	208 (4.34)	31.1	70.2
21	3	4	1 (0.01)	0(0)	1 (0.03)	2 (0.04)	50	25
Total	5800	29187	7618 (36.73)	13120 (63.27)	3887 (46.01)	4562 (53.99)	39.4	71.1

Data includes the number of children observed, number of visits made to Pingilikani dispensary, the number and proportion of malaria among ITN use or non-ITN-users in the 21 geographical areas

**Figure 3.**
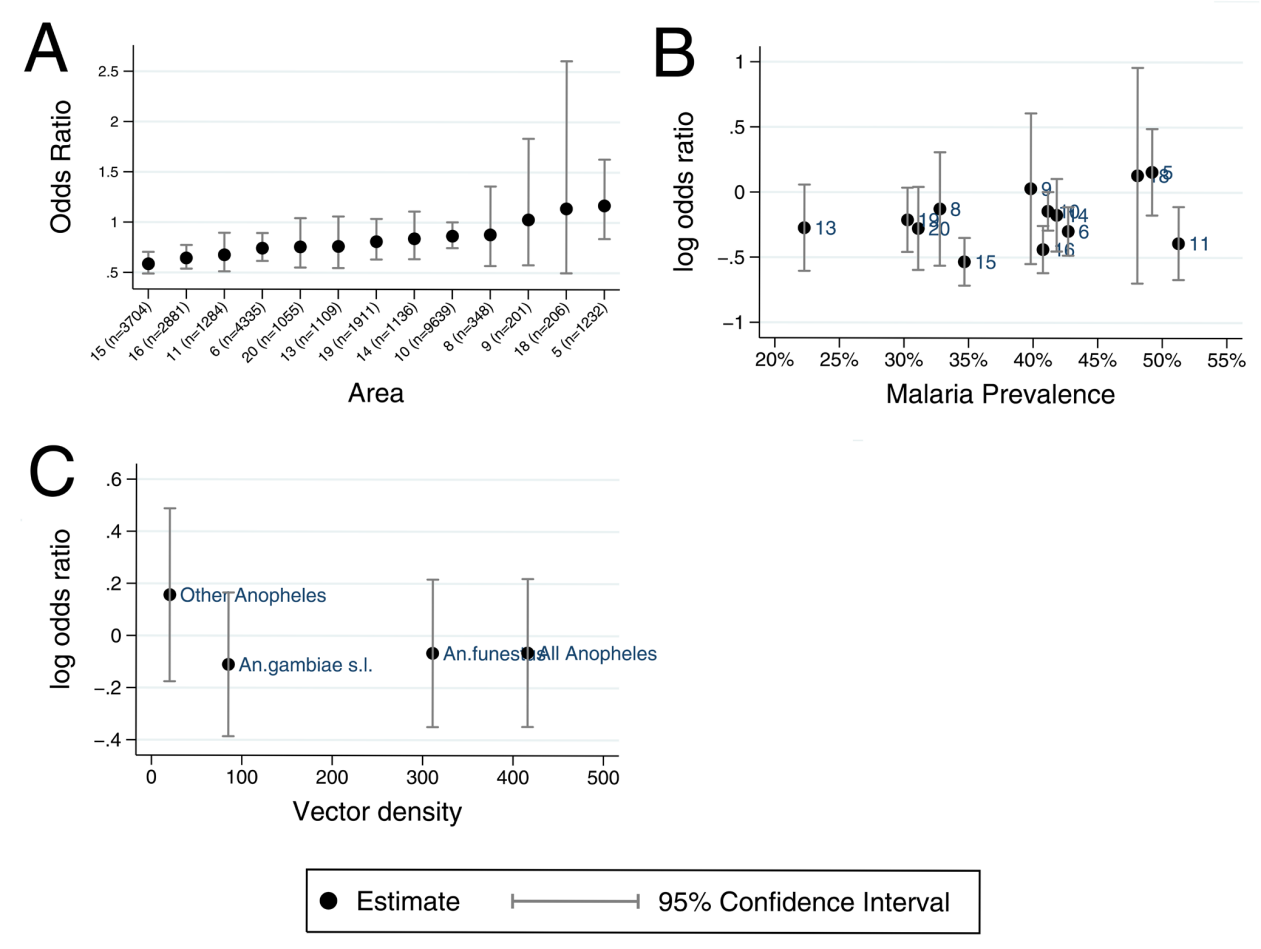
Panel A shows a scatter plot of stratum specific adjusted Odds Ratio of insecticide treated net (ITN) effect in 13 geographical areas in order of decreasing effectiveness. Panel **B** shows a scatter plot of the log odds ratio of ITN effect against malaria prevalence in 13 geographical areas. Panel **C** shows a scatter plot of the log odds ratio of ITN effect against overall mosquito densities and for the 3 separate taxa.

Among children who were ITN users, 37% (7618/20738) of the visits were associated with positive malaria slides, whereas among non-ITN-users 46% (3887/8449) of the visits were associated with positive malaria slides. ITN use was associated with a 32% protection from malaria; crude OR = 0.68, 95% CI: 0.64, 0.73 (p<0.001). When geographical area was added to the model as an interaction term with ITN use, we obtained a variation in ITN effectiveness between the geographical areas (p=0.0055). Geographical variation in ITN effectiveness remained robust (p=0.016) even after adjusting for the year of visitation to the dispensary, season and the interactions between ITN use and nonlinear age ( Supplementary Table 1). The stratum specific adjusted OR for the association of ITN use on malaria in the geographical areas was calculated and shown in the order of decreasing effectiveness ( Figure 3A). Previous data have shown that ITN effectiveness is lower in areas of high malaria transmission ^11, 45^. This did not appear to be the explanation for variation in effectiveness in this data ( Figure 3B); the Spearman rho coefficient value for the association of ITN effectiveness and prevalence of malaria was 0.1868, p=0.541.

### Hotspots

**Figure 4.**
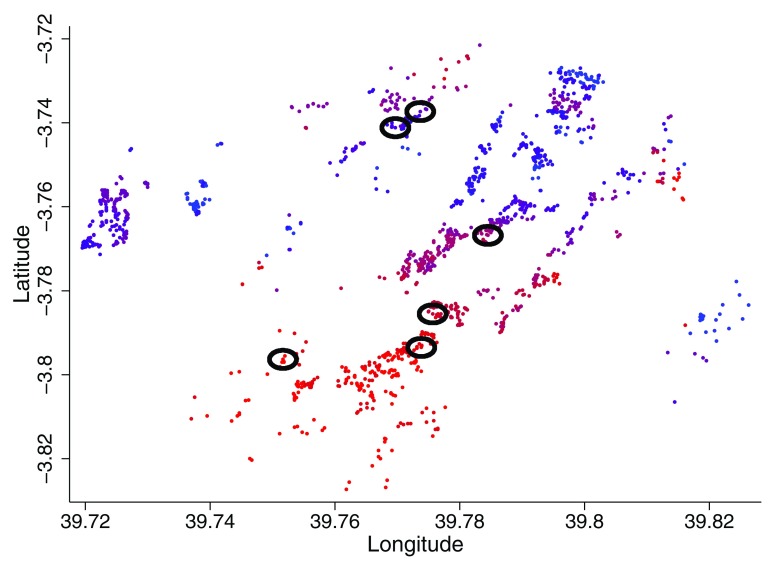
Scatter plot of estimated insecticide treated net (ITN) effectiveness for individual homesteads aggregated at a 2.5km smoothing. Each plotted point represents an individual homestead, where color shading indicates ITN effectiveness, with red shading indicating low effectiveness and blue shading indicating high effectiveness. The large black circles indicate the significant hotspots (analyzed without smoothing).

Using the logistic regression model, we estimated ITN effectiveness for each individual homestead where there was sufficient data to calculate a point estimate (i.e. >30 observations from homestead aggregated at a 2.5 km smoothing). Using SaTScan software, we identified 6 hotspots of low ITN effectiveness: p=0.001 for the 6 hotspots ( Figure 4). We concluded that spatial variation in ITN effectiveness was not due to random noise based on the 95% confidence intervals obtained from the logistic regression analysis for geographical areas and the existence of hotspots by SaTScan, and selected six geographical areas for further entomological studies to represent a range of ITN effectiveness estimates.

### Vector abundance

Over 26 nights, 415 female *Anopheles* mosquitoes were collected by both methods (i.e. 272 by HLC and 143 by CDC-LT), representing a mean of 16 mosquitoes per night. 66% of mosquitoes were collected using HLC. Of the 415 mosquitoes, 311 (75%) were *An. funestus* group, 84 (20%) were *An. gambiae s.l.* and 20 (5%) were other *Anopheles* i.e. *An. pretoriensis, An. coustani, An. moucheti and An. squamosus* ( Table 2). The *An. funestus* group was caught more than *An. gambiae s.l* (p<0.001). Of the 84 amplified samples of *An. gambiae s.l*., 68 (81%) were *An. Arabiensis* and 16 (19%) were *An. gambiae s.s*. The proportion of *Anopheles* mosquitoes caught outdoors (60%; 95% CI: 55%, 65%) was greater than the proportion caught indoors (p<0.001). There were more *Anopheles* mosquitoes collected outdoors in all geographical areas except area 6, where most of the mosquitoes were collected indoors ( Table 2). The frequencies of vectors collected in each geographical area are summarized in Supplementary Table 2. *An. funestus* group was the most prevalent vector in all areas. However, we did not find an association between ITN effectiveness and vector density, Spearman rho coefficient was -0.2, p=0.8 ( Figure 3C). Of the 272 mosquitoes collected by HLC, 3.3% (9/272) tested positive for *P. falciparum* sporozoites. The most detected sporozoite infectious mosquitoes captured were from the *An. funestus* group (7/9). The rate of indoor and outdoor biting estimated by HLC was 19.8 and 25.5 bites per person per night, respectively.

**Table 2.** Proportion of *Anopheles* mosquitoes collected indoors and outdoors by either HLC or CDC-LT.

	Number collected	n (%) Indoor	n (%) Outdoor	ITN effectiveness (CI)
**All**	415	165 (39.8%)	250 (60.2%)	
**Vectors**				
*Anopheles arabiensis*	68	7 (10.3%)	61 (89.7%)	
*Anopheles coustani*	6	0	6 (100%)	
*Anopheles funestus group*	311	152 (48.9%)	159 (51.1%)	
*Anopheles gambiae s.s.*	16	6 (37.5%)	10 (62.5%)	
*Anopheles moucheti*	1	0	1 (100%)	
*Anopheles pretoriensis*	12	0	12 (100%)	
*Anopheles squamosus*	1	0	1 (100%)	
**Geographical area**				
**5**	192	89 (46.4%)	103 (53.6%)	-16.9 [-6.3, 16.1]
**19**	105	12 (11.4%)	93 (88.6%)	19.1 [-0.4, 36.8]
**20**	47	12 (25.5%)	35 (74.5%)	24.2 [-0.4, 44.9]
**6**	60	50 (83.3%)	10 (16.7%)	25.8 [1.1, 38.4]
**15**	5	1 (20.0%)	4 (80.0%)	35.5 [2.3, 46.2]
**16**	6	1 (16.7%)	5 (83.3%)	41.3 [3.0, 51.1]

area 5, 19 and 20 were regarded as low effectiveness area; area 6, 15 and 16 were regarded as high effectiveness area; CI: Confidence Interval; %: Proportion per 100

**Figure 5.**
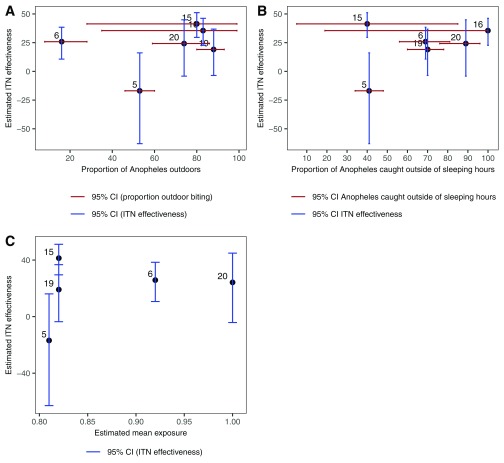
Panel **A** shows a scatter plot of estimated insecticide treated net (ITN) effectiveness and the proportion of *Anopheles* mosquitoes collected outdoors. Panel **B** shows a scatter plot of ITN effectiveness against the proportion of *Anopheles* mosquitoes caught outside of sleeping hours (i.e. < 23:00hrs and > 5:00hrs). Panel **C** shows a scatter plot of ITN effectiveness against the estimated mean exposure π _s_.

The frequency and proportion of *Anopheles* mosquitoes collected in the six areas of high vs. low ITN effectiveness are summarised in Table 3 and Supplementary Figure 1. Overall, the proportion of mosquitoes caught outdoor was higher in the low ITN effectiveness areas (67% vs. 27%, p <0.001), but this apparent significance was due to a single area (labelled area 6), which was an outlier for mosquitoes caught indoor ( Figure 5A). When we excluded area 6, the proportion of mosquitoes caught outdoor in the low vs. high ITN effectiveness areas was non-significant (67% vs. 82% p=0.306). Moreover, when we analysed the proportion of mosquitoes caught outside of sleeping hours, <23:00hrs and > 5:00hrs, by individual geographical area there was not a visually obvious trend with decreasing ITN effectiveness in the six geographical areas ( Figure 5B), although this association could have been limited by the power of the study, as evidenced by the confidence intervals. The Spearman rho coefficient value for the association of ITN effectiveness and proportion of mosquitoes collected outdoors was 0.1429, p=0.79.

**Table 3.** Composition of the *Anopheles* mosquito vector in areas of high and low ITN effectiveness.

Trap type	Vectors	Low ITN effectiveness areas			High ITN effectiveness area		
		Total (N)	Outdoor (n)	Outdoor (%)	Total (N)	Outdoor (n)	Outdoor (%)
HLC	*Anopheles arabiensis*	48	42	87.5	5	4	80.0
	*Anopheles coustani*	6	6	100.0	0	0	0.0
	*Anopheles funestus group*	163	70	42.9	23	10	43.5
	*Anopheles gambiae s.s.*	13	7	53.8	0	0	0.0
	*Anopheles moucheti*	1	1	100.0	0	0	0.0
	*Anopheles pretoriensis*	12	12	100.0	0	0	0.0
	*Anopheles squamosus*	1	1	100.0	0	0	0.0
CDC-LT	*Anopheles arabiensis*	15	15	100.0	0	0	0.0
	*Anopheles coustani*	0	0	0.0	0	0	0.0
	*Anopheles funestus group*	82	74	90.2	43	5	11.6
	*Anopheles gambiae s.s.*	3	3	100.0	0	0	0.0
	*Anopheles moucheti*	0	0	0.0	0	0	0.0
	*Anopheles pretoriensis*	0	0	0.0	0	0	0.0
	*Anopheles squamosus*	0	0	0.0	0	0	0.0

*HLC: Human landing catches; CDC-LT: CDC light trap, %: Proportion per 100, N & n: number of mosquitoes collected.

### Human behaviour

**Figure 6.**
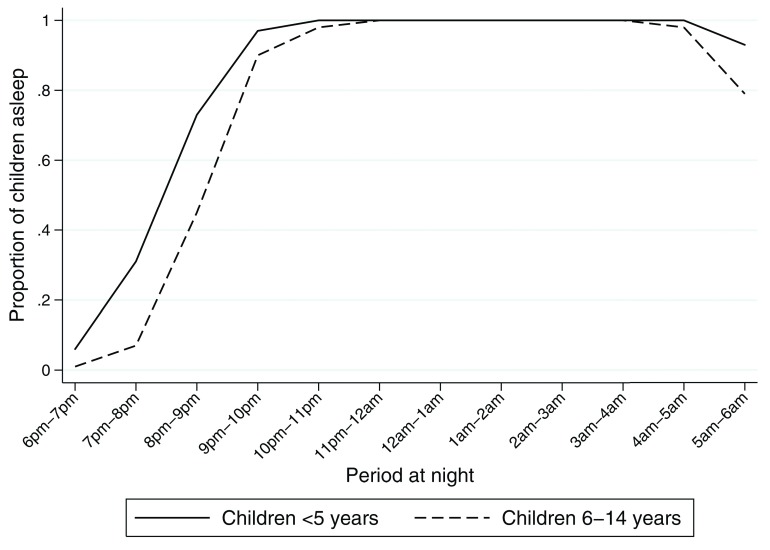
Proportion of children asleep at each hour of the night.

**Figure 7.**
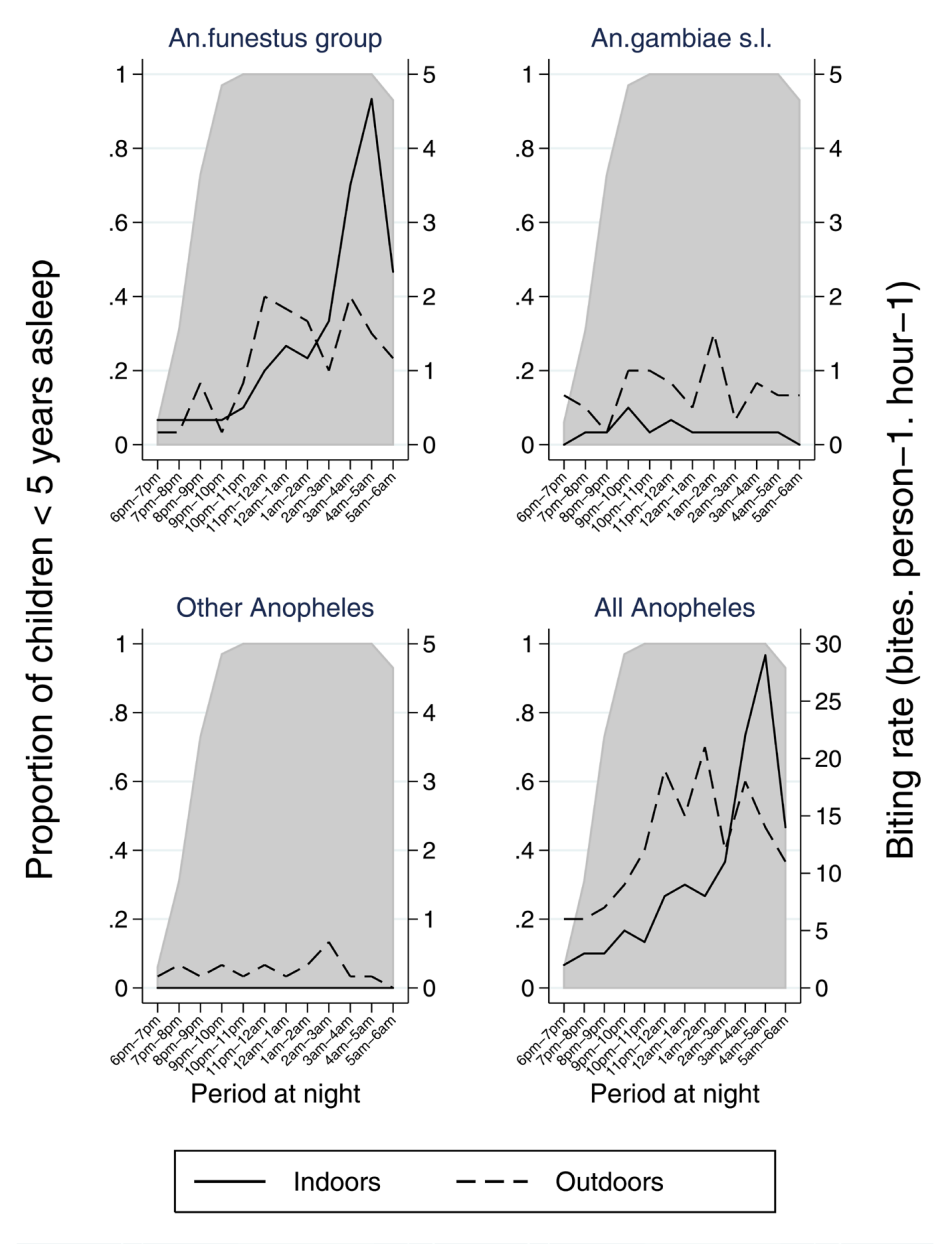
Hourly biting pattern of Anopheles mosquitoes occurring both indoors (solid lines) and outdoors (dashed lines) for the 3 separate taxa and overall. The grey area represents the proportion of the children < 5 years asleep at each hour of the night.

**Figure 8.**
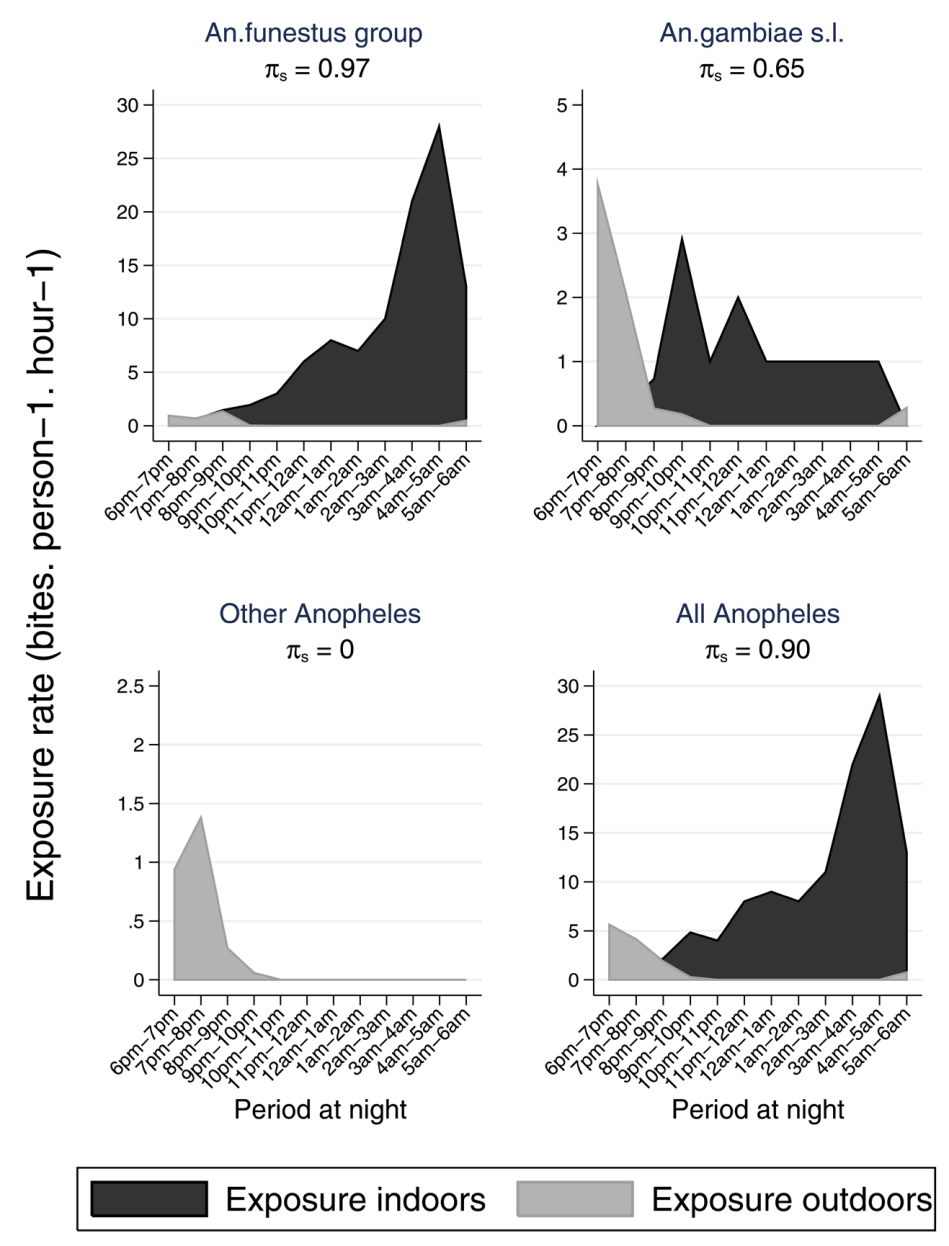
Estimated mean exposure indoor and outdoor for 3 separate taxa and for all Anopheles in the six geographical areas among children <5 years.

Seventy three percent of children <5 years were reported to be asleep between 6 pm and 9 pm, these rose monotonically over the course of the night reaching 100% by 10 pm ( Table 4 & Figure 6). A similar trend was observed in areas of high and low ITN effectiveness ( Supplementary Table 3 & Supplementary Table 4). Children aged between 6–14 years spent more time awake, only 45% were asleep before 9 pm ( Figure 6 & Supplementary Table 5). The timing of human activity and sleeping behaviour in particular modulates the effect of human-mosquito contact and the effectiveness of ITN. We quantified the interaction between mosquitoes and humans to evaluate whether outdoor vector biting is a potential explanation for the variation in ITN effectiveness. The peak biting activity for each mosquito vector is illustrated in Figure 7. Clearly higher indoor biting activity was observed among the *An. funestus* group. The overall propensity to feed at times when most people were asleep was high in the *An. funestus* group and *An. gambiae s.l.*, except for other *Anopheles* ( Figure 8): the vast majority of the *Anopheles* mosquitoes were caught at times when most people are indoors asleep ( Figure 7). Estimates for the proportion of human-mosquito contact between the first and last hour when most humans were asleep was consistently high across all locations, ranging from 0.83 to 1.00 ( Figure 5C). The estimated proportion of exposure to *Anopheles* mosquito bites that occurred indoor was high.

**Table 4.** Estimated fraction of human exposure to mosquito bites occurring indoor and outdoor among children <5 years using Equation 1 overall.

Time of the night	Proportion of children <5 years asleep	Mosquitoes caught indoors	Mosquitoes caught outdoors	Weighted mean indoor biting rates by the proportion of children <5 years reporting to be asleep	Weighted mean outdoor biting rates by the proportion of children <5 years not yet asleep	Estimation of the fraction of human exposure which LLIN can realistically confer direct personal protection	Estimation of the fraction of human exposure which occurs outdoors
6pm–7pm	0.06	2	6	0.12	5.64	0.02	0.98
7pm–8pm	0.31	3	6	0.93	4.14	0.18	0.82
8pm–9pm	0.73	3	7	2.19	1.89	0.54	0.46
9pm–10pm	0.97	5	9	4.85	0.27	0.95	0.05
10pm–11pm	1.00	4	12	4.00	0.00	1.00	0.00
11pm–12am	1.00	8	20	8.00	0.00	1.00	0.00
12am–1am	1.00	9	15	9.00	0.00	1.00	0.00
1am–2am	1.00	8	20	8.00	0.00	1.00	0.00
2am–3am	1.00	11	12	11.00	0.00	1.00	0.00
3am–4am	1.00	22	18	22.00	0.00	1.00	0.00
4am–5am	1.00	29	14	29.00	0.00	1.00	0.00
5am–6am	0.93	14	11	13.02	0.77	0.94	0.06
Total (π _s_)		118	150	112.11	12.71	0.90	0.10

Assuming sleeping time = time indoor (this gives the lower bound fraction human exposure that can be reduced by LLINs)

## Discussion

Malaria transmission has reduced dramatically over the last 15 years in Kilifi, evidenced by falling rates of clinical malaria cases in hospital ^31, 32^, in the community ^46^ and falling community prevalence of asymptomatic infection ^47^. A recent resurgence has been noted with increasing cases among older children, and increasing prevalence of infection more widely around the coast ^31, 34^. The reductions have been temporally associated with marked reductions in the prevalence of the abundance of vectors ^22^ and with a pronounced shift away from *Anopheles gambiae s.s*, which was previously the dominant vector, and towards *Anopheles arabiensis* in terms of relative abundance. In addition, many countries, including Kenya, have attempted to reduce this burden by increasing ITN ownership and usage ^48, 49^. However, previous reports have shown that prolonged ITN use leads to behavioral shifts in the mosquito vector from indoor to outdoor biting or feeding in the early part of the evening ^6, 22, 27, 50^. This shift in mosquito feeding behavior might be expected to limit the effectiveness of ITNs. We identified geographical variation in the effectiveness of ITN and identified areas where ITN effectiveness was found to be consistent with the 50% estimate reported in the literature ^11, 51, 52^, and other areas where ITNs were less effective ( Figure 3A). This variation could conceivably have arisen as a result of variations in quality of ITNs, the physical integrity of ITNs, patterns of use, host resistance, insecticide resistance, bioefficacy of the insecticidal compounds or other factors, including random variation. We sought to investigate whether variations in outdoor vector biting was a potential explanation.

We found that *An. funestus* group was more prevalent than *An. gambiae s.l.* species complex, consistent with previous report ^22^. Among *An. gambiae s.l.* species complex, *An. arabiensis* was more prevalent, which is known to be capable of feeding extensively on humans early in the evenings, before humans go indoors ^17, 53^. This shift in sibling species composition has previously been reported ^6, 22^. We observed small-scale spatial variability in vector abundance ( Table 2), which is consistent with previous reports on the Kenyan Coast ^20, 54^. We also observed a higher proportion of mosquito vectors collected outside of sleeping hours, in areas of both high and low ITN effectiveness ( Figure 5B). On first principles one would expect that outdoor exposure would limit ITNs effectiveness. However, despite seeing more mosquitoes caught outside of sleeping hours throughout the study area this did not appear to be associated with an overall reduction in ITN effectiveness. The trend towards outdoor exposure was of modest epidemiological significance and is within the normal range of variation for these vectors ^55^. The captured microheterogeneity of the estimated mean exposure or mosquitoes caught outside of sleeping hours does not clearly explain the microheterogeneity in ITN effectiveness ( Figure 5A–C). We may have observed an apparently statistically significant increase in the abundance of mosquitoes caught outdoor in areas of low ITN effectiveness. However, this was due to a single outlying geographical area and there was no variation in abundance of mosquitoes caught outdoor after this area was excluded. This suggests the statistical significance of the initial comparison may have been due to ecological confounding, where a geographical area with high ITN effectiveness happened to have more indoor mosquitoes, but this relationship was not confirmed in other areas ( Figure 5A). We also did not find a clear role of either vector in driving the heterogeneity observed ( Figure 3C & Supplementary Figure 2).

It is possible that the higher proportion of mosquitoes caught outdoors/outside of sleeping hours represents a behavioral response to unsuccessful feeding attempts made indoors during the night, and therefore it may simply be a marker of successful ITN use. This avoidance behavior may exert a cost on the vector, and so ITNs may in fact still be protective in areas where outdoor exposure is observed, as has been suggested previously ^30^. Furthermore, outdoor exposure and the probability of successful feeding outside of sleeping hours cannot be directly inferred from the human landing catches, since the landing catches are not in themselves sufficient to survey pattern of normal human exposure to mosquito bite. Once adjusted for human behaviour, most human-vector interaction in this study occurred indoors ( Figure 8). Outdoor exposure is currently not a major factor influencing residual malaria transmission since 95% of the population are indoors at the peak biting period for malaria vector mosquitoes. Human behaviour is the primary driver of when and where exposure occurs and is far more variable than the mosquito behaviour that matter within a single vector species ^55^.

Spatial heterogeneity in malaria exposure has been described at micro-epidemiological level at varying transmission settings ^56^ and is responsible for variations in disease risk within small geographical areas and is evidenced by local clustering of malaria infections. The observed geographical variation in ITN effectiveness therefore remains unexplained. Possibilities include insecticide resistance, or geographical variations in human behaviour in terms of ITN use. While it is also possible that non-linearity in the relationship between transmission intensity and clinical episodes could explain the variations in ITN effectiveness, we did not identify a consistent relationship between ITN effectiveness and transmission intensity ( Figure 3B). Furthermore we identified statistical evidence of effect modification between geographical location and ITN effectiveness (p=0.016), suggesting that lack of power in selected geographical locations is unlikely to be the explanation for variation.

Our study has some limitations. Data on ITN use may have been incorrectly reported, as we did not require each resident to be present during the survey. We attempted to minimize this by instructing data collecting teams to interview only residents of the same homestead regarding ITN ownership and usage. There may have been some misclassification as we did not ascertain ITN use during hospital presentation but instead used the yearly ITN data collected by the annual survey. The results may also be biased and confounded by other unmeasured factors (e.g., variation in the quality and type of ITN, urbanization, socio-economic status and mother’s education). It is likely that we underestimated the protection afforded by the use of high-quality ITN because we included all ITNs, regardless of quality, physical integrity or bioefficacy of the insecticidal compounds. The vast majority of ITNs in the area are long-lasting insecticidal nets, hence we do not expect substantial variation in insecticidal efficacy. The accuracy of the mosquito survey is limited by the practical challenges of maintaining consistently sensitive human landing catches throughout the night. Lack of explicit molecular data for distinguishing sibling species and molecular forms within the *An. funestus* group introduces ambiguity into the interpretation of the results of the study. In addition boiling and retesting CSP could be done to increase specificity of the ELISA results. In this study, we examined variations in the personal protection afforded by ITNs and did not examine variation in community level effect. The size of our study limits power: with a sample size of 415, and the proportion of mosquitoes biting outdoors at 67% in low ITN effectiveness areas we therefore had >90% power to detect a reduction to 27% or lower in high ITN effectiveness areas. Our study was therefore powered to detect only a large difference in the proportion of vectors caught outdoors. However, we reasoned that reductions of ITN effectiveness to less than half of the previously documented efficacy of 50% would require a doubling of the proportion of mosquitoes feeding outdoors. Hence our study was powered to detect large variations in the frequency of outdoor exposure. In addition, the accuracy of mosquito sampling data is limited as only one month of sampling was conducted in this study, we recommend sampling for a longer duration of time.

In summary, our data do not provide evidence of an epidemiological association between microgeographical variations in ITN effectiveness and variations in the microgeographical distribution of outdoor exposure. The outdoor exposure observed may therefore have been the result of high levels of ITN use leading to unsuccessful attempts at indoor feeding. However, it remains possible that continued selection pressures might lead to the emergence of populations of mosquitoes that are better adapted to outdoor feeding in the future. Outdoor feeding is becoming more common in parts of Africa ^57^ and may represent evolutionary change in some areas, with a potential to undermine ITN effectiveness. With outdoor fractions of transmission being so low, and individual human behavior being so heterogenous, it may be expected to be epidemiologically detectable only once indoor transmission has been more effectively tackled and individual-level estimates of exposure distributions are measured ^19, 58^. Therefore, malaria control programs require monitoring to assess the impact of ITNs on vector populations and vector behavioral change as well as monitoring ITN effectiveness as vectors evolve ^6, 23, 26– 28^. Continuous monitoring of vector bionomics, and malaria transmission dynamics are essential for predicting disease outbreaks and guiding vector control in the region. Furthermore, capacity needs to be built in interpreting and applying these data to malaria control policy.

## Ethical approval

This study was approved by the Kenya Medical Research Institute Scientific Ethics Review Unit (KEMRI/SERU/CGMR-C/024/3148). Written informed consent was obtained from the parents/guardians of the children attending the dispensary.

## Data availability

Data that support the findings of this study (hospital surveillance, ITN community surveys and mosquito collection) are available from the KEMRI Institutional Data Access/Ethics Committee, for researchers who meet the criteria for access to confidential data. Details of the criteria can be found in the KEMRI-Wellcome data sharing guidelines. The data includes homestead level coordinates as an essential component and these are personally identifiable data. Access to data is provided via the KEMRI-Wellcome Data Governance Committee: Data_Governance_Committee@kemriwellcome.org; Tel, +254708 587 210; Contact person, Marianne Munene (Secretary; Tel, +254709 983 436).

## Supplementary material

**Supplementary Table 1: The odds ratio and 95% CI of the univariate and multivariate logistic regression with robust standard errors.** Data includes the stratum specific adjusted Odds Ratio (aOR) and the Confidence Interval (95% CI); ‡ areas with fewer than 35 observations were excluded from the logistic regression due to perfect prediction and/or collinearity.

**Supplementary Table 2: Composition of the mosquito species in six geographical areas.** Data includes number of *Anopheles* mosquitoes collected by human landing catches (HLC) and CDC light trap (CDC-LT) indoor or outdoor in the six geographical areas, and the overall proportion.

**Supplementary Table 3: Estimated fraction of human exposure to mosquito bites occurring indoor and outdoor among children <5 years using Equation 1 in the high ITN effectiveness areas.**

**Supplementary Table 4: Estimated fraction of human exposure to mosquito bites occurring indoor and outdoor among children <5 years using Equation 1 in the low ITN effectiveness areas.**

**Supplementary Table 5: Estimated fraction of human exposure to mosquito bites occurring indoor and outdoor among children 6–14 years using Equation 1 overall.**

**Supplementary Figure 1: Bar graph of the proportion of *Anopheles* mosquito species collected in areas of low and high insecticide treated net (ITN) effectiveness.**

**Supplementary Figure 2: A scatter plot of malaria prevalence and the densities of each taxon.**
