# Supplementary figures and images for: Variation in the effectiveness of insecticide treated nets against malaria and outdoor biting by vectors in Kilifi, Kenya
Source: Wellcome Open Res. 2018 Dec 3;2:22. Originally published 2017 Mar 30. [Version 4] doi: 10.12688/wellcomeopenres.11073.4 (PMC6281023; doi:10.12688/wellcomeopenres.11073.4)

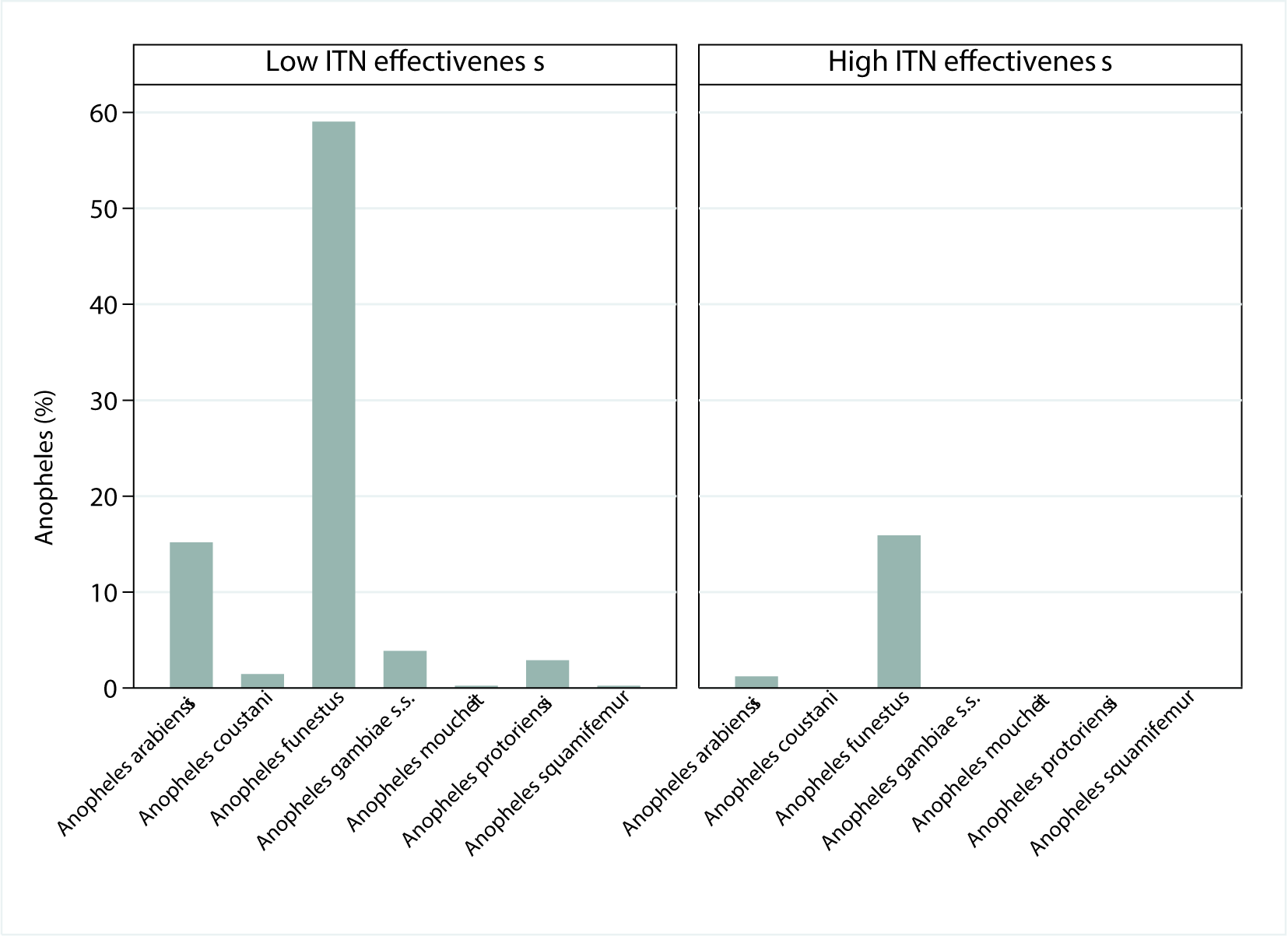

Supplement: Supplementary file 6 [file wellcomeopenres-2-16305-s0005.tgz › ebc3f9c0-e45f-4621-8369-5bbca9e77a56_New_supplementary_fig_1.tif]

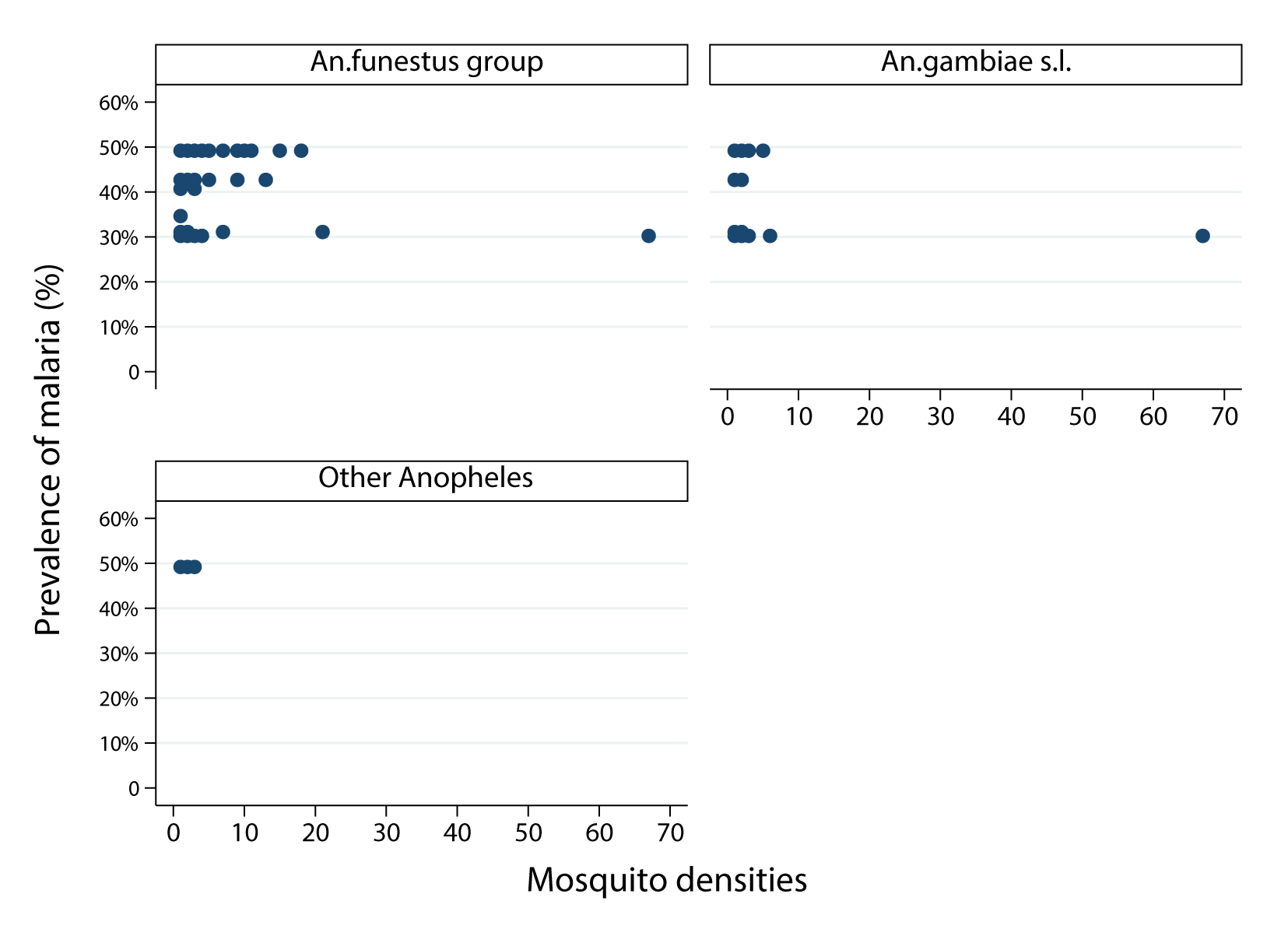

Supplement: Supplementary file 7 [file wellcomeopenres-2-16305-s0006.tgz › b2ba57f4-c987-49ad-824e-597b3e42d2be_New_supplementary_fig_2.tif]
