# Supplementary material for: Variation in the effectiveness of insecticide treated nets against malaria and outdoor biting by vectors in Kilifi, Kenya
Source: Wellcome Open Res. 2018 Dec 3;2:22. Originally published 2017 Mar 30. [Version 4] doi: 10.12688/wellcomeopenres.11073.4 (PMC6281023; doi:10.12688/wellcomeopenres.11073.4)
Supplement: Peer review history

# Peer review: Referee response for version 4

**DOI:** 10.21956/wellcomeopenres.16305.r34367

**Published:** 2018-12-04

**Authors:** Gerry F Killeen

**Affiliations:** 1 Environmental Health and Ecological Sciences Thematic group, Ifakara Health Institute, Dar es Salaam, Tanzania

While I might be tempted to mention the potential role of vector taxonomic heterogeneity (eg varying composition of the An. funestus group) as a possible determinant of effectiveness heterogeneities, this is otherwise this is a very fine piece of work and very balanced in terms of interpretation. The authors are to be congratulated for their rigour, commitment and persistence.

I have read this submission. I believe that I have an appropriate level of expertise to confirm that it is of an acceptable scientific standard.

# Peer review: Referee response for version 3

**DOI:** 10.21956/wellcomeopenres.16269.r34232

**Published:** 2018-11-14

**Authors:** Gerry F Killeen

**Affiliations:** 1 Environmental Health and Ecological Sciences Thematic group, Ifakara Health Institute, Dar es Salaam, Tanzania

OVERALL

 This revision is a vast improvement, reflecting an impressive commitment by the authors to getting the science right and making the most of this important study. Still some important opportunities for improvement, however, so I provide some substantive but hopefully final comments. I know this has been a long, tough road for the authors on a very detailed manuscript, but I’d encourage them to give this one last solid revision.

 MAJOR COMMENTS

 While the Y axis label of figure 8 looks correct, the numbers plotted look like the proportion of exposure for each hour rather than the absolute rate of exposure. The latter is what is needed (columns 5 and 6 from table 4) and will make this figure not only more accurate, but also far more appealing and easy to digest. The current version, based on columns 3 and 4 of table 2 is misleading and over-represents the significance of the outdoor biting in the early evening because they are lower, so the relative size of the areas under these two stacked lines don’t match the estimate for π _s _at the top of the graph which is correct. Just use columns 5 and 6, and this will look very nice and match well to both the numerical estimates of proportional exposure in table 4 and similar graphs and estimates from elsewhere in Africa.

 Like figure 7, figure 8 should include separate panels for different Anopheles taxa. Given that there’s so few PCR-identified gambiae and no clear difference in behaviour obvious in the figure 7 graphs, I would tend to merge An. arabiensis and An. gambiae ss into a single panel for An. gambiae complex. I would also retain the An funestus group panel and other Anopheles panel, but add All Anopheles as the fourth panel. The taxonomic breakdown of the 4 panels in figures 7 and 8 should be the same, so that the two figures are consistent and readily comparable. With columns 5 and 6 of table 4 (or equivalent for that particular taxon) being used for all the plots in figure 8, it will also be easier to get exposure rates to the 4 different vector taxa into clearer perspective as the biting rates are in the current figure 7. I think supplementary figure 3 probably contains the required content (albeit in need of correction for exposure rate rather than proportion), but I couldn’t open this EPS format and suggest this kind of material belongs in the main manuscript.

 For similar reasons, I would like to also see numbers for An. gambiae sl in table 2. Incidentally, I would find table 2 easier to read if the percentages came before n, or were presented with one decimal place of precision and/or a % sign.

 What you’ve calculated is indeed π _s_ rather than π _i_ , and that was the right choice in this case, so you should be consistent in how you describe it in words throughout the narrative: “..while asleep..” rather than “.. while indoors..” as per the abstract, and perhaps we more specific about the assumptions underlying the interpretation elsewhere in the text: “..while asleep, presumably indoors..”?

 I like supplementary figure 1 and would suggest including in the main manuscript. In fact, wouldn’t it render the current figure 3 redundant, or make a nice second panel in that figure? I’d also like to see additional panels (and associated statistical tests) in this figure with An. funestus sl density, An gambiae sl density, Other Anopheles, and All Anopheles as the X-axes.  

 An additional figure with plots of prevalence versus densities of each taxon would also be valuable, and can be put in the supplementary material if nothing interesting is obvious. While it’s nice to look at behaviours, perhaps the simplest explanation that has been observed elsewhere is variations in vector density.

 Figure 5 and associated text is very useful, but the proportion of mosquitoes caught outdoors (exophagy) is not usually the driver of unpreventable human exposure, but rather the proportion caught outside of sleeping hours (crepuscularity)-See Huho 2013. So I’d recommend adding a panel to this figure with this perhaps more relevant metric on the x-axis, as well as a third for overall π _i_ capturing the combined outcome of both parameters in each location. On that theme, the narrative “The statistical trend towards outdoor biting was of modest epidemiological significance…” is accurate but needs clearer explanation for the average reader.

 This interpretational misalignment is also reflected in the discussion, a few lines from the end of page 13: “..Our study was therefore powered to detect only a large difference in the proportion of vectors caught outdoors.”. Please digest Huho et al 2013 carefully and then rephrase to give the proportions caught during/outside of sleeping hours at least as much emphasis. Similarly, the opening sentence of page 14 “outdoor biting” might be better rephrased as “outdoor exposure”, and similar phrases throughout that closing paragraph also carefully adjusted for accuracy and clarity?

 Final paragraph of background: Be aware of the fact that An. arabiensis (not “arabienses”) exhibits at least three behaviours that can enable residual transmission: In addition to feeding outdoors (usually achieved by biting early), it can also survive by attacking animals or through repeated, cautious foraging inside houses until an unprotected non-user is found. See the background section of Malaria Journal 15: 255 for a brief overview.

 While I know I suggested that “It is also possible that nonlinearity in the relationship between transmission intensity and clinical episodes explains the variations in ITN effectiveness.”, I think supplementary figure 1 rules out that explanation, so you should say so and this is just one more reason to move this into the main text as an extra panel in figure 3.

 In the closing paragraph of the discussion, I’d suggest the following additional words: “WITH outdoor fractions of transmission BEING SO LOW, AND INDIVIDUAL HUMAN BEHAVIOUR BEING SO HETEROGENOUS, it MAY be expected to be epidemiologically detectable ONLY once indoor transmission has been MORE EFFECTIVELY tackled AND INDIVIDUAL-LEVEL ESTIMATES OF EXPOSURE DISTRIBUTIONS ARE MEASURED.” For the last point, cite Msellemu 2016 and Bradley 2015.

 SUNDRY MINOR CORRECTIONS AND SUGGESTIONS

 Closing sentence of the results, suggest “was consistently high ACROSS ALL LOCATIONS…”.

 Third sentence, first paragraph of discussion: Looks like a simple editing mistake. Also best to avoid suggesting and increase in absolute rather than just relative abundance of An. arabiensis. I suggest: “…a pronounced shift away from Anopheles gambiae s.s, which was previously the dominant vector, and TOWARDS Anopheles arabiensis IN TERMS OF RELATIVE ABUNDANCE.”

 End of first paragraph on page 10, a minor grammatical error: “….sought to investigate whether…” rather than “…sought to investigateD whether..”

 The terms “significant”, “significantly different” etc are rather over-used redundantly. In scientific writing, one doesn’t state any difference unless it’s statistically significant so the statement that one thing is greater or lesser than another or that there’s a trend infers significance unless stated otherwise, and the P value provided in brackets, table etc adds the evidence to support such a statement of difference or trend.

 When talking about absolute mosquito densities per se, I think the terms “abundant”/”abundance” and more appropriate than “prevalence”/”prevalent”, which suggests a proportion. Suggest correcting throughout.

 Without boiling and retesting for CSP, the ELISA results are a little questionable ^1^, especially for any zoophagic non-vectors or secondary vectors, and this study limitation should be acknowledged.

I have read this submission. I believe that I have an appropriate level of expertise to confirm that it is of an acceptable scientific standard, however I have significant reservations, as outlined above.

## References

[1] DurnezLVan BortelWDenisLRoelantsPVeracxATrungHDSochanthaTCoosemansM: False positive circumsporozoite protein ELISA: a challenge for the estimation of the entomological inoculation rate of malaria and for vector incrimination. *Malar J*.2011;10: 10.1186/1475-2875-10-195 195 10.1186/1475-2875-10-195 21767376PMC3160429

# Response

**Published:** 2018-11-27

**Authors:** Alice Kamau

**Affiliations:** KEMRI-Wellcome Trust Research Programme, Kenya

Dear Dr. Gerry F. Killeen,

We are very grateful to Prof. Killen for reviewing our manuscript, “Variation in the effectiveness of insecticide treated nets against malaria and outdoor biting by vectors in Kilifi, Kenya” and providing insightful and useful comments this far. We respond below to the points raised, indicating the reviewer’s points with “Q” and our responses by “A” in bold.

**Reviews /comments:**

**Specific Questions**

Q1) While the Y axis label of figure 8 looks correct, the numbers plotted look like the proportion of exposure for each hour rather than the absolute rate of exposure. The latter is what is needed (columns 5 and 6 from table 4) and will make this figure not only more accurate, but also far more appealing and easy to digest. The current version, based on columns 3 and 4 of table 2 is misleading and over-represents the significance of the outdoor biting in the early evening because they are lower, so the relative size of the areas under these two stacked lines don’t match the estimate for π at the top of the graph which is correct. Just use columns 5 and 6, and this will look very nice and match well to both the numerical estimates of proportional exposure in table 4 and similar graphs and estimates from elsewhere in Africa.

**A1: We have adjusted the figure accordingly, see New Figure 8.**

Q2) Like figure 7, figure 8 should include separate panels for different Anopheles taxa. Given that there’s so few PCR-identified gambiae and no clear difference in behaviour obvious in the figure 7 graphs, I would tend to merge An. arabiensis and An. gambiae ss into a single panel for An. gambiae complex. I would also retain the An funestus group panel and other Anopheles panel, but add All Anopheles as the fourth panel. The taxonomic breakdown of the 4 panels in figures 7 and 8 should be the same, so that the two figures are consistent and readily comparable. With columns 5 and 6 of table 4 (or equivalent for that particular taxon) being used for all the plots in figure 8, it will also be easier to get exposure rates to the 4 different vector taxa into clearer perspective as the biting rates are in the current figure 7. I think supplementary figure 3 probably contains the required content (albeit in need of correction for exposure rate rather than proportion), but I couldn’t open this EPS format and suggest this kind of material belongs

in the main manuscript.

**A2: We have adjusted the figures accordingly, see New Figure 7 and New Figure 8.**

Q3) For similar reasons, I would like to also see numbers for An. gambiae sl in table 2. Incidentally, I would find table 2 easier to read if the percentages came before n, or were presented with one decimal place of precision and/or a % sign.

**A3: We have added the % sign and presented the % to one decimal place. Table 2 contains** **data obtained from the ELISA-CSP and molecular analysis** **with the mosquitoes differentiated to species except for *An. funestusgroup* which we do not have molecular data on.**

Q4) What you’ve calculated is indeed π rather than π , and that was the right choice in this case, so you should be consistent in how you describe it in words throughout the narrative: “..while asleep..” rather than “.. while indoors..” as per the abstract, and perhaps we more specific about the assumptions underlying the interpretation elsewhere in the text: “..while asleep, presumably indoors..”?

**A4: We have made revision as shown below:**

**Abstract section**

**The overall propensity to feed at times when most people were asleep was high; the vast majority of the Anopheles mosquitoes were caught at times when most people are indoors asleep. Estimates for the proportion of human-mosquito contact between the first and last hour when most humans were asleep was consistently high across all locations, ranging from 0.83 to 1.00.**

**Result section**

**The overall propensity to feed at times when most people were asleep was high in the *An. funestus* group and *An. gambiae s.l.*, except for other *Anopheles* (Figure 8): the vast majority of the *Anopheles* mosquitoes were caught at times when most people are indoors asleep (Figure 7). Estimates for the proportion of human-mosquito contact between the first and last hour when most humans were asleep was consistently high across all locations, ranging from 0.83 to 1.00 (Figure 5C).** **The estimated proportion of exposure to *Anopheles* mosquito bites that occurred indoor was high.**

Q5) I like supplementary figure 1 and would suggest including in the main manuscript. In fact, wouldn’t it render the current figure 3 redundant, or make a nice second panel in that figure? I’d also like to see additional panels (and associated statistical tests) in this figure with An. funestus sl density, An gambiae sl density, Other Anopheles, and All Anopheles as the X-axes.

**A5: We have made revision as shown below:**

**We have moved supplementary figure 1 to the main manuscript as a second panel in figure 3 i.e. Figure 3B and added Figure 3C that shows a scatter plot of the odds ratio of insecticide treated net (ITN) effect and 95% confidence interval of malaria positivity against overall** **mosquito densities and for the 3 separate taxa.**

**Result section**

***An. funestus* group was the most prevalent vector in all areas. However, we did not find an association between ITN effectiveness and vector density, Spearman rho coefficient was -0.2, p=0.8 (Figure 3C).**

**Discussion section**

**We also did not find a clear role of either vector in driving the heterogeneity observed (Figure 3C &** **Supplementary Figure 2).**

Q6) An additional figure with plots of prevalence versus densities of each taxon would also be valuable, and can be put in the supplementary material if nothing interesting is obvious. While it’s nice to look at behaviours, perhaps the simplest explanation that has been observed elsewhere is variations in vector density.

**A6: We have made revision as shown below:**

**An additional figure with plots of prevalence vs densities of each taxon has been added as Supplementary Figure 2.**

**Discussion section**

**We also did not find a clear role of either vector driving the heterogeneity observed (Supplementary Figure 2).**

Q7) Figure 5 and associated text is very useful, but the proportion of mosquitoes caught outdoors (exophagy) is not usually the driver of unpreventable human exposure, but rather the proportion caught outside of sleeping hours (crepuscularity)-See Huho 2013. So I’d recommend adding a panel to this figure with this perhaps more relevant metric on the x-axis, as well as a third for overall π capturing the combined outcome of both parameters in each location. On that theme, the narrative “The statistical trend towards outdoor biting was of modest epidemiological significance…” is accurate but needs clearer explanation for the average reader.

**A7: We have addressed the above comment as follows:**

**We have added a graph that shows ITN effectiveness against the proportion of anopheles caught outside of sleeping hours i.e. Figure 5B.**

**We have also added a graph that shows ITN effectiveness against the estimated mean exposure π _s. _i.e. Figure 5C.**

**Result section**

**Moreover, when we analysed the proportion of mosquitoes caught outside of sleeping hours, <23:00hrs and > 5:00hrs, by individual geographical area there was not a visually obvious trend with decreasing ITN effectiveness in the six geographical areas (Figure 5B), although this association could have been limited by the power of the study, as evidenced by the confidence intervals. The Spearman rho coefficient value for the association of ITN effectiveness and proportion of mosquitoes collected outdoors was 0.1429, p=0.79.**

**Discussion section**

**However, despite seeing more mosquitoes caught outdoor throughout the study area this did not appear to be associated with an overall reduction in ITN effectiveness. The trend towards outdoor exposure was of modest epidemiological significance and is within the normal range of variation for these vectors [55]. The captured microheterogeneity of the estimated mean exposure or mosquitoes caught does not clearly explain the microheterogeneity in ITN effectiveness (Figure 5A-C). We may have observed an apparently statistically significant increase in the abundance of mosquitoes caught outdoor in areas of low ITN effectiveness. However, this was due to a single outlying geographical area and there was no variation in abundance of mosquitoes caught outdoor after this area was excluded. This suggests the statistical significance of the initial comparison may have been due to ecological confounding, where a geographical area with high ITN effectiveness happened to have more indoor mosquitoes, but this relationship was not confirmed in other areas (Figure 5A & B). We also did not find a clear role of either vector in driving the heterogeneity observed (Figure 3C &** **Supplementary Figure 2).**

Q8) This interpretational misalignment is also reflected in the discussion, a few lines from the end of page 13: “..Our study was therefore powered to detect only a large difference in the proportion of vectors caught outdoors.”. Please digest Huho et al 2013 carefully and then rephrase to give the proportions caught during/outside of sleeping hours at least as much emphasis. Similarly, the opening sentence of page 14 “outdoor biting” might be better rephrased as “outdoor exposure”, and similar phrases throughout that losing paragraph also carefully adjusted for accuracy and clarity?

**A8:  We have rephrased as follows:**

**We also observed a higher proportion of mosquito vectors collected outside of sleeping hours, in areas of both high and low ITN effectiveness (Figure 5B). On first principles one would expect that outdoor exposure would limit ITNs effectiveness. However, despite seeing more mosquitoes caught outside of sleeping hours throughout the study area this did not appear to be associated with an overall reduction in ITN effectiveness. The trend towards outdoor exposure was of modest epidemiological significance and is within the normal range of variation for these vectors [55]. The captured microheterogeneity of the estimated mean exposure or mosquitoes caught outside of sleeping hours does not clearly explain the microheterogeneity in ITN effectiveness (Figure 5A-C).**

**It is possible that the higher proportion of mosquitoes caught outdoors/outside of sleeping hours represents a behavioral response to unsuccessful feeding attempts made indoors during the night, and therefore it may simply be a marker of successful ITN use. This avoidance behavior may exert a cost on the vector, and so ITNs may in fact still be protective in areas where outdoor exposure is observed, as has been suggested previously [30]. Furthermore, outdoor exposure and the probability of successful feeding outside of sleeping hours cannot be directly inferred from the human landing catches, since the landing catches are not in themselves sufficient to survey pattern of normal human exposure to mosquito bite. Once adjusted for human behaviour, most human-vector interaction in this study occurred indoors (Figure 8).**

**In summary, our data do not provide evidence of an epidemiological association between microgeographical variations in ITN effectiveness and variations in the microgeographical distribution of outdoor exposure. The outdoor exposure observed may therefore have been the result of high levels of ITN use leading to unsuccessful attempts at indoor feeding.**

Q9) Final paragraph of background: Be aware of the fact that An. arabiensis (not “arabienses”) exhibits at least three behaviours that can enable residual transmission: In addition to feeding outdoors (usually achieved by biting early), it can also survive by attacking animals or through repeated, cautious foraging inside houses until an unprotected non-user is found. See the background section of Malaria Journal 15: 255 for a brief overview.

**A9: We have made revision in the background section shown below:**

**In Western Kenya and South-eastern Tanzania there have been reports of a reduction in indoor feeding by *An. gambiae sensu stricto ( s.s.)* and an increase in the relative abundance of *An. arabiensis.* The latter has a broader range of feeding times and biting behavior, including: feeding at dusk or dawn on humans outdoors; readily feeding on animals when available; or repeatedly foraging inside houses until an unprotected non-ITN user is found [8, 17, 23, 25, 26].**

Q10) While I know I suggested that “It is also possible that nonlinearity in the relationship between transmission intensity and clinical episodes explains the variations in ITN effectiveness.”, I think supplementary figure 1 rules out that explanation, so you should say so and this is just one more reason to move this into the main text as an extra panel in figure 3.

**A10: We have made revisions accordingly in the discussion as shown below:**

**While it is also possible that non-linearity in the relationship between transmission** i **ntensity and clinical episodes could explain the variations in ITN effectiveness, we did not identify a consistent relationship between ITN effectiveness and transmission intensity (Figure 3B).**

Q11) In the closing paragraph of the discussion, I’d suggest the following additional words: “WITH outdoor fractions of transmission BEING SO LOW, AND INDIVIDUAL HUMAN BEHAVIOUR BEING SO HETEROGENOUS, it MAY be expected to be epidemiologically detectable ONLY once indoor transmission has been MORE EFFECTIVELY tackled AND INDIVIDUAL-LEVEL ESTIMATES OF EXPOSURE DISTRIBUTIONS ARE MEASURED.” For the last point, cite Msellemu 2016 and Bradley 2015.

**A11: We have made the revision accordingly.**

Q12) Closing sentence of the results, suggest “was consistently high ACROSS ALL LOCATIONS…”.

**A12:  We have made the revision accordingly.**

Q13) Third sentence, first paragraph of discussion: Looks like a simple editing mistake. Also best to avoid suggesting and increase in absolute rather than just relative abundance of An. arabiensis. I suggest: “…a pronounced shift away from Anopheles gambiae s.s, which was previously the dominant vector, and TOWARDS Anopheles arabiensis IN TERMS OF RELATIVE ABUNDANCE.”

**A13:  We have made revisions accordingly.**

Q14) End of first paragraph on page 10, a minor grammatical error: “….sought to investigate whether…” rather than “…sought to investigateD whether..”

**A14: We have made the revision accordingly.**

Q15) The terms “significant”, “significantly different” etc are rather over-used redundantly. In scientific writing, one doesn’t state any difference unless it’s statistically significant so the statement that one thing is greater or lesser than another or that there’s a trend infers significance unless stated otherwise, and the P value provided in brackets, table etc adds the evidence to support such a statement of difference or trend.

**A15: We have made revisions accordingly.**

Q16) When talking about absolute mosquito densities per se, I think the terms “abundant”/”abundance” and more appropriate than “prevalence”/”prevalent”, which suggests a proportion. Suggest correcting throughout.

**A16: We have made revisions accordingly.**

Q17) Without boiling and retesting for CSP, the ELISA results are a little questionable , especially for any zoophagic non-vectors or secondary vectors, and this study limitation should be acknowledged.

**A17: We have included this under the limitation as follows:**

**In addition boiling and retesting CSP could be done to increase specificity of the ELISA results.**

# Peer review: Referee response for version 2

**DOI:** 10.21956/wellcomeopenres.14871.r29812

**Published:** 2018-01-29

**Authors:** Gerry F Killeen

**Affiliations:** 1 Environmental Health and Ecological Sciences Thematic group, Ifakara Health Institute, Dar es Salaam, Tanzania

OVERALL

 This version of the manuscript reflects a lot of hard and meticulous work by the authors, and is vastly improved with additional, highly relevant data brought to bear, plus much more appropriate analysis. However, some aspects of the analysis, and even more so the interpretation, still need some substantive work. As it stands, there are substantial limitations to what the data and analysis can tell us, and the lack of any detectable association between measures of outdoor exposure and measures of impact are presented far too conclusively as evidence of lack of underlying association.

 MAJOR COMMENTS

 My most important reservations about the results and especially the discussion section are best captured in the conclusions section of the abstract, which is unjustified when stated as follows:

 “Our data therefore do not support the hypothesis that outdoor biting limits the effectiveness of ITNs in our study area.”

 Actually, the entomological and human behaviour data do. For a start, the mean of about 10% of transmission occurring outdoors is consistent with studies from all across Africa [1], but that estimate assumes no protection from a bed net. Adjusting for the protective effects of bed nets, that means about 50% of remaining exposure to residual transmission occurs outdoors for bed net users [2, 3]. So these results are very consistent with the persistence of malaria transmission, even amongst bed net users, and the contribution of outdoor transmission to that situation.

 Indeed, the epidemiological data do not provide evidence of an epidemiological association between malaria prevalence and variations in the distribution of human exposure occurring outdoors. However, there are a number of potential explanations for this other than lack of an underlying relationship:

1. The most obvious biological factor that could explain variations in ITN effectiveness is baseline transmission. Malaria burden responds non-linearly to transmission exposure with areas experiencing higher reinfection rates being less responsive to transmission control because the human population becomes saturated with infection. Immunity also plays its part in dampening the relationship, by easing off as control is more effective. However, I don’t think this is the explanation in this case because…
2. The most obvious statistical reason for such variations in ITN effectiveness lie in detectability, and therefore run in exactly the opposite direction. Stronger effects could be seen in areas with more malaria cases, where effect sizes may be easier to quantify. It is noticeable that the largest confidence intervals in Figure 3 are consistently associated with the lowest estimates of protection. Unlikely to be a coincidence. Also, in Table 3, the most obvious difference between the putative high and low ITN effectiveness areas is that the latter have very few mosquitoes, so probably much less transmission and burden to begin with. ITNs cannot protect people against malaria they are not be exposed to.
3. The non-random pattern in Figure 4 looks consistent with either possibility too. I would therefore like to see Figure 3 ordered by overall prevalence/incidence rather than by OR, and an analysis to test that relationship carried out.
4. Figure 5 confirms by best guess that the biggest limitation to reliably proving any such association lies in measurement error.
5. There are many other potential confounders, the most important of which is probably composition of the Anopheles funestus group. Let’s remember that Anopheles parensis was first discovered as a species on the Kenyan coast where it was found biting outdoors in the early evening but did not transmit appreciable levels of malaria. I note that the Anopheles funestus group was not identified to species level, so all these variations in mosquito behaviour and human exposure patters could be explained by variations in the abundance of much weaker vectors within the group that feed more outdoors at dawn and dusk. Reference 38 only covers PCR methods for the An. gambiae complex and reference 6 is inappropriate, indirect and doesn’t cover the specific methodology required.

I conclude that final sentence of the abstract is too stark and over-represents the significance of the lack of any evidence for an association, which is not the same thing as evidence of a lack of association.

 Figure 8 over-represents the importance of outdoor biting species that are less effective vectors than indoor biting vectors by pooling them into a single figure, resulting in the kind of double-peak that is typical of these graphs where two or more species are undifferentiated-we discussed this a weakness of our own An. funestus group data in our Africa-wide review [1]. Please provide 4 separate panels for the 4 separate taxa. Please be explicit about what is really species specific, and what reflects potentially two or more species from a complex or group. Just to be transparent, use the term “An. funestus group” throughout.

 In many places, the existing evidence base is misrepresented and the wording needs to be edited to be more precise and avoid exaggerating the case being made. In addition to all the following specific comments, please read and digest more carefully the previously provided references.

 For example, in the middle of the second paragraph of the Introductions on page 3: “..which could be caused by changes in the behaviour of the…” suggests these behaviours are new phenomena. It is a common mistake to represent such phenomena as something new, rather than something we previously ignored but are now becoming more conscious of. While heritable changes in mosquito behaviour do appear to be occurring in some well-studied locations, the overwhelmingly point of all our previous review work has been that outdoor feeding has always occurred but wasn’t recognized as a problem, because we hadn’t done a good enough a job of killing mosquitoes indoors. We therefore hadn’t reached the stage we are at now, where that outdoor transmission is now an important component of our much smaller remaining transmission problem. While reference 22 is a reasonably convincing example where a potentially heritable behaviour change within a single species appears to be happening, this is the exception and all the other studies cited are equivocal because there are several alternative explanations [2, 3, 7].

 MINOR COMMENTS

 The phraseology is also a little loose later in the Introduction where the term “shift” in the relative abundance of species is used to hint at species replacement, imply potential for in a “decrease in ITN effectiveness” rather than merely a limitation of ITN effectiveness. Actually, this probably reflects differential partial success rather than any literal undermining of impact [7].

 In the results section, the statistical trend towards outdoor biting is of modest epidemiological significance and its within the normal range of variation for these vectors [1].

 At the end of the second paragraph of the introduction, the definition of exophagy is over-extended: “…., ie the vector feeds outdoors on humans” implies exophagy combined with anthropophagy, rather than just exophagy.

 Opening sentence of third paragraph of the introduction: The Anopheles funestus group is a group, not a complex so the word “taxa” should be used instead of “species complex” at the start of the sentence.

 Third sentence, first paragraph of introduction: Is the at-risk population referred to global or African? Please be specific. Also say estimated, rather than modelled, as lots of real data were used and the latter term can turn off non-specialists.

 In the third paragraph of the introduction, references 6 and 27 are from southern Tanzania, not northern Tanzania.

 In the results section, towards the end of the vector abundance subsection, the term “rate” is used rather loosely: “Higher sporozoite rate was observed among the An. funestus group (7/9).” I think what your are trying to say is that most detected sporozoite inoculations/infectious mosquitoes captured were from the An. funestus group.

 Looks like a typo or endnote error at the end of the first sentence of the second paragraph in the discussion.

 A little overwritten in places, for example:

 Replace “optimally effective” with “most effective” at the start of the second paragraph of the Introduction section.

I have read this submission. I believe that I have an appropriate level of expertise to confirm that it is of an acceptable scientific standard, however I have significant reservations, as outlined above.

## References

[1] HuhoBBriëtOSeyoumASikaalaCBayohNGimnigJOkumuFDialloDAbdullaSSmithTKilleenG: Consistently high estimates for the proportion of human exposure to malaria vector populations occurring indoors in rural Africa. *Int J Epidemiol*.2013;42(1) : 10.1093/ije/dys214 235-47 10.1093/ije/dys214 23396849PMC3600624

[2] KilleenGF: A second chance to tackle African malaria vector mosquitoes that avoid houses and don't take drugs. *Am J Trop Med Hyg*.2013;88(5) : 10.4269/ajtmh.13-0065 809-16 10.4269/ajtmh.13-0065 23589532PMC3752742

[3] KilleenGF: Characterizing, controlling and eliminating residual malaria transmission. *Malar J*.2014;13: 10.1186/1475-2875-13-330 330 10.1186/1475-2875-13-330 25149656PMC4159526

[4] SmithDLDushoffJSnowRWHaySI: The entomological inoculation rate and Plasmodium falciparum infection in African children. *Nature*.2005;438(7067) : 10.1038/nature04024 492-5 10.1038/nature04024 16306991PMC3128496

[5] BeierJCKilleenGFGithureJI: Short report: entomologic inoculation rates and Plasmodium falciparum malaria prevalence in Africa. *Am J Trop Med Hyg*.1999;61(1) :109-13 1043206610.4269/ajtmh.1999.61.109

[6] GilliesM.TFurlongM: An investigation into the behaviour of Anopheles parensis Gillies at Malindi on the Kenya coast. *Bulletin of Entomological Research*.1964;55(1) :1-16 10.1017/S0007485300049221

[7] GovellaNJChakiPPKilleenGF: Entomological surveillance of behavioural resilience and resistance in residual malaria vector populations. *Malar J*.2013;12: 10.1186/1475-2875-12-124 124 10.1186/1475-2875-12-124 23577656PMC3637503

# Response

**Published:** 2018-11-05

**Authors:** Alice Kamau

**Affiliations:** KEMRI-Wellcome Trust Research Programme, Kenya

Dear Dr. Gerry F. Killeen,

Thank you for reviews of our manuscript, “Variation in the effectiveness of insecticide treated nets against malaria and outdoor biting by vectors in Kilifi, Kenya”. We respond below to the points raised, indicating the reviewer’s points with “Q” and our responses by “A” in bold.

**Reviews /comments:**

**Specific Questions**

Q1) Indeed, the epidemiological data do not provide evidence of an epidemiological association between malaria prevalence and variations in the distribution of human exposure occurring outdoors. However, there are a number of potential explanations for this other than lack of an underlying relationship: The most obvious biological factor that could explain variations in ITN effectiveness is baseline transmission. Malaria burden responds non-linearly to transmission exposure with areas experiencing higher reinfection rates being less responsive to transmission control because the human population becomes saturated with infection. Immunity also plays its part in dampening the relationship, by easing off as control is more effective. However, I don’t think this is the explanation in this case because…

**A1: We have included this potential explanation in the discussion as follows:**

**It is possible that non-linearity in the relationship between transmission intensity and clinical episodes explains the variations in ITN effectiveness.  However we did not identify a consistent relationship between ITN effectiveness and transmission intensity (Supplementary figure 1).**

Q2) The most obvious statistical reason for such variations in ITN effectiveness lie in detectability, and therefore run in exactly the opposite direction. Stronger effects could be seen in areas with more malaria cases, where effect sizes may be easier to quantify. It is noticeable that the largest confidence intervals in Figure 3 are consistently associated with the lowest estimates of protection. Unlikely to be a coincidence. Also, in Table 3, the most obvious difference between the putative high and low ITN effectiveness areas is that the latter have very few mosquitoes, so probably much less transmission and burden to begin with. ITNs cannot protect people against malaria they are not be exposed to.

**A2: Supplementary Figure 1 shows no consistent variation.  We have references this in the text:**

**Furthermore we identified statistical evidence of effect modification between geographical location and ITN effectiveness (p=0.016), suggesting that lack of power in selected geographical locations is unlikely to be the explanation for variation.**

Q3) The non-random pattern in Figure 4 looks consistent with either possibility too. I would therefore like to see Figure 3 ordered by overall prevalence/incidence rather than by OR, and an analysis to test that relationship carried out.

**A3: The log odds ratio in Supplementary Figure 1 were ordered by malaria prevalence and the Spearman rho coefficient value for the association of ITN effectiveness and prevalence of malaria was 0.1868, p=0.541 as shown below. We had included this in the result section as shown below:**

**Previous data have shown that ITN effectiveness is lower in areas of high malaria transmission [11, 45]. This did not appear to be the explanation for variation in effectiveness in this data. (Supplementary Figure 1); the Spearman rho coefficient value for the association of ITN effectiveness and prevalence of malaria was 0.1868, p=0.541.**

Q4) Figure 5 confirms by best guess that the biggest limitation to reliably proving any such association lies in measurement error.

**A4: We have made revision in the results section as shown below:**

**Moreover, when analysed by individual geographical area there was not a visually obvious trend associating mosquitoes caught outdoor biting with decreasing ITN effectiveness in the six geographical areas ( Figure 5), although this association could have been limited by the power of the study, as evidenced by the confidence intervals shown in Figure 5.**

Q5) There are many other potential confounders, the most important of which is probably composition of the *Anopheles funestus* group. Let’s remember that *Anopheles parensis* was first discovered as a species on the Kenyan coast where it was found biting outdoors in the early evening but did not transmit appreciable levels of malaria. I note that the *Anopheles funestus* group was not identified to species level, so all these variations in mosquito behaviour and human exposure patters could be explained by variations in the abundance of much weaker vectors within the group that feed more outdoors at dawn and dusk. Reference 38 only covers PCR methods for the *An. gambiae* complex and reference 6 is inappropriate, indirect and doesn’t cover the specific methodology required.

**A5: We have included appropriate reference and delete reference 6 from the methodology as shown below:**

 Koekemoer LL, Kamau L, Hunt RH, Coetzee M: **A cocktail polymerase chain reaction assay to identify members of the Anopheles funestus (Diptera: Culicidae) group.** *Am J Trop Med Hyg *2002, **66:**804-811.

**We do not have molecular data for the *An. funestus* group. We had included this as a limitation in the discussion section as shown below**

**Discussion section**

**Lack of explicit molecular data for distinguishing sibling species and molecular forms within the *An. funestus* group introduces ambiguity into the interpretation of the results of the study**

Q6) I conclude that final sentence of the abstract is too stark and over-represents the significance of the lack of any evidence for an association, which is not the same thing as evidence of a lack of association.

**A6: We have made revision of the final sentence of the abstract as follows:**

**Our data do not provide evidence of an epidemiological association between microgeographical variations in ITN effectiveness and variations in the microgeographical distribution of outdoor biting.**

Q7) Figure 8 over-represents the importance of outdoor biting species that are less effective vectors than indoor biting vectors by pooling them into a single figure, resulting in the kind of double-peak that is typical of these graphs where two or more species are undifferentiated-we discussed this a weakness of our own *An. funestus* group data in our Africa-wide review. Please provide 4 separate panels for the 4 separate taxa. Please be explicit about what is really species specific, and what reflects potentially two or more species from a complex or group. Just to be transparent, use the term “ *An. funestus *group” throughout.

**A7: We have addressed the above comment as follows:**

**We have added Supplementary Figure 3 which provides the estimated mean exposure indoor and outdoor in four separate panels for the 4 separate taxa**

**We have revised the result section as shown below:**

**The overall propensity to feed at times when most people are indoor was high overall (Figure 8) and in the *An. funestus* group and *An. gambiae s.l*.,** **except for *An. Arabiensis* and other *Anopheles*** **(Supplementary Figure 3): the vast majority of the *Anopheles* mosquitoes were caught at times when most people are indoor (Figure 7).**

Q8) For example, in the middle of the second paragraph of the Introductions on page 3: “..which could be caused by changes in the behaviour of the…” suggests these behaviours are new phenomena. It is a common mistake to represent such phenomena as something new, rather than something we previously ignored but are now becoming more conscious of. While heritable changes in mosquito behavior do appear to be occurring in some well-studied locations, the overwhelmingly point of all our previous review work has been that outdoor feeding has always occurred but wasn’t recognized as a problem, because we hadn’t done a good enough a job of killing mosquitoes indoors. We therefore hadn’t reached the stage we are at now, where that outdoor transmission is now an important component of our much smaller remaining transmission problem. While reference 22 is a reasonably convincing example where a potentially heritable behaviour change within a single species appears to be happening, this is the exception and all the other studies cited are equivocal because there are several alternative explanations.

**A8:  We have rephrased as follows:**

**However, residual malaria transmission is well described even after optimal ITN use, which could be associated with outdoor biting behaviour of the mosquito vector that allows them to evade fatal contact with these frontline tools of intervention [17, 18].**

Q9) The phraseology is also a little loose later in the Introduction where the term “shift” in the relative abundance of species is used to hint at species replacement, imply potential for in a “decrease in ITN effectiveness” rather than merely a limitation of ITN effectiveness. Actually, this probably reflects differential partial success rather than any literal undermining of impact [7].

**A9: We have made the revision accordingly.**

**Both species complexes feed primarily indoors; however, both have exhibited outdoor biting or feeding in the early part of the evening in some areas where ITNs have been deployed [6, 19-22].**

Q10) In the results section, the statistical trend towards outdoor biting is of modest epidemiological significance and is within the normal range of variation for these vectors [1].

**A10: We have made revisions accordingly in the discussion.**

**We also observed a higher proportion of mosquito vectors collected outdoors than indoors, in areas of both high and low ITN effectiveness ( Figure 5). On first principles one would expect that outdoor biting would lead to ITNs becoming ineffective. However, despite seeing more mosquitoes caught outdoor throughout the study area this did not appear to be associated with an overall reduction in ITN effectiveness. We may have observed an apparently statistically significant increase in the prevalence of mosquitoes caught outdoor in areas of low ITN effectiveness. However, this was due to a single outlying geographical area and there was no variation in prevalence of mosquitoes caught outdoor after this area was excluded. This suggests the statistical significance of the initial comparison may have been due to ecological confounding, where a geographical area with high ITN effectiveness happened to have more indoor mosquitoes, but this relationship was not confirmed in other areas ( Figure 5). The statistical trend towards outdoor biting is of modest epidemiological significance and it is within the normal range of variation for these vectors [55].**

Q11) At the end of the second paragraph of the introduction, the definition of exophagy is over-extended: “…., ie the vector feeds outdoors on humans” implies exophagy combined with anthropophagy, rather than just exophagy.

**A11: We have made the revision accordingly.**

**The most obvious behavioural change is the mosquito vector exhibiting exophagic tendencies –i.e. the vector feeds outdoors.**

Q12) Opening sentence of third paragraph of the introduction: The *Anopheles funestus* group is a group, not a complex so the word “taxa” should be used instead of “species complex” at the start of the sentence.

**A12:  We have made the revision accordingly.**

**Among malaria vectors in Africa, the two principal taxa are: *Anopheles gambiaesensu lato (s.l.)* and *Anopheles funestus* group.**

Q13) Third sentence, first paragraph of introduction: Is the at-risk population referred to global or African? Please be specific. Also say estimated, rather than modelled, as lots of real data were used and the latter term can turn off non-specialists.

**A13:  We have made revisions accordingly.**

**Estimates from model-based predictions suggest that approximately 1.4 billion of the global population live at risk of stable malaria and ~1.1 billion at risk of unstable malaria.**

Q14) In the third paragraph of the introduction, references 6 and 27 are from southern Tanzania, not northern Tanzania.

**A14: We have made the revision accordingly.**

**In southern Tanzania, where ITNs have been used for several years, the mosquitoes are biting more frequently during the hours of the early evening and early morning when people are more likely to be awake and vulnerable outside of their nets [6,27].**

Q15) In the results section, towards the end of the vector abundance subsection, the term “rate” is used rather loosely: “Higher sporozoite rate was observed among the *An. funestus* group (7/9).” I think what your are trying to say is that most detected sporozoite inoculations/infectious mosquitoes captured were from the *An. funestus* group.

**A15: We have made revisions accordingly.**

**The most detected sporozoite infectious mosquitoes captured were from the *An. funestus* group (7/9).**

Q16) Looks like a typo or endnote error at the end of the first sentence of the second paragraph in the discussion.

**A16: It was a typo and we have revised accordingly.**

Q17) A little overwritten in places, for example: Replace “optimally effective” with “most effective” at the start of the second paragraph of the Introduction section.

**A17: We have made revision in the introduction as suggested.**

**The frontline tools for malaria control in sub-Saharan Africa, insecticide treated nets (ITNs) and indoor residual spray, are most effective if baseline transmission occurs indoors**

# Peer review: Referee response for version 1

**DOI:** 10.21956/wellcomeopenres.11943.r23190

**Published:** 2017-06-02

**Authors:** Sarah J. Moore

**Affiliations:** 1 Swiss Tropical and Public Health Institute, Basel, Switzerland; 2 Ifakara Health Institute, Bagamoyo Research and Training Centre, Bagamoyo, Tanzania

The authors have conducted a study addressing the hypothesis that outdoor feeding of mosquitoes undermines the effectiveness of ITNs.

 The entomology presented in the paper is inadequate to answer the hypothesis presented for the following reasons:

1. 5 years of clinical data are presented (2009-2014) but only one month of mosquito sampling is conducted in 2016, two years after the last piece of clinical data was collected.
2. No PCR speciation was reported. In this area there are a number of cryptic species that look the same but differ in both their behaviour and their ability to transmit malaria. No molecular techniques were used to test the mosquito species. So you could have a switch from *An. gambiae s.s.* that bites indoors and has high vectorial competence to *An. arabiensis *that bites outdoors and has lower vectorial competence. The same is true in the *An. funestus *complex that is comprised of a number of outdoor biting species like *An. leesoni *or *An. rivulorum.*
3. The authors reported that PCR (polymerase chain reaction) was done on the mosquitoes yet I cannot find data in the paper reporting the outcome of the PCR. All data reports *An. gambiae *s.l. and *An. funestus *group.

The paper explores changing mosquito behaviour with lowered effectiveness of nets but only used one month of vector collections two years after the clinical data was collected to test this link and the actual species present are not reported. I therefore find this a big stretch of the data. Vector density, composition and behaviour varies throughout the year and these collections were made for a short time. I therefore don’t think the data are sufficient to accept or reject the hypothesis. 

 That being said the rest of the data is very useful and nicely presented. The data do demonstrate that there is substantial outdoor biting in June/July, and I should like to see the species composition in the area seeing as the authors report that the PCR was done. Outdoor biting may not increase malaria if the vectors doing the outdoor biting are not very competent for malaria.

I have read this submission. I believe that I have an appropriate level of expertise to confirm that it is of an acceptable scientific standard, however I have significant reservations, as outlined above.

# Response

**Published:** 2018-01-06

**Authors:** Alice Kamau

**Affiliations:** KEMRI-Wellcome Trust Research Programme, Kenya

We are grateful for this review and the helpful comments and suggestions that have been made. We have included a point-by-point response (in bold) to the issues raised.

Q1) 5 years of clinical data are presented (2009-2014) but only one month of mosquito sampling is conducted in 2016, two years after the last piece of clinical data was collected.

**A1: We have updated the clinical surveillance data to December 2016 and updated the manuscript accordingly.**

Q2) No PCR speciation was reported. In this area there are a number of cryptic species that look the same but differ in both their behaviour and their ability to transmit malaria. No molecular techniques were used to test the mosquito species. So you could have a switch from An. gambiae s.s. that bites indoors and has high vectorial competence to An. arabiensis that bites outdoors and has lower vectorial competence. The same is true in the An. funestus complex that is comprised of a number of outdoor biting species like An. leesoni or An. rivulorum.

**A2: We have** **included data obtained from the ELISA-CSP and molecular analysis** **in the results section. The mosquitoes were differentiated to species as shown under the result section. However, we do not have molecular data for the *An. funestus group*. We have included this as a limitation in the discussion section as shown below**

**Result section**

**“Over 26 nights, 415 female *Anopheles* mosquitoes were collected by both methods (i.e. 272 by HLC and 143 by CDC-LT), representing a mean of 16 mosquitoes per night. 66% of mosquitoes were collected using HLC. Of the 415 mosquitoes morphologically identified, 311 (75%) were *An. funestus group*, 84 (20%) were *An. gambiae s.l.* and 20 (5%) were other Anopheles i.e. *An. protoriensis, An. coustani, An. moucheti and An. squamosus* (Table 2). The An. funestus group was significantly greater than An. gambiae s.l (p<0.001). Out of the 84 amplified samples of *An. gambiae s.l*., 68 (81%) were *An. Arabiensis* and 16 (19%) were *An. gambiae s.s.* The proportion of *Anopheles* mosquitoes caught outdoors (60%; 95% CI: 55%, 65%) was significantly greater than the proportion caught indoors (p<0.001). There were more *Anopheles* mosquitoes collected outdoors in all geographical areas except area 6, where most of the mosquitoes were collected indoor (Table 2). The frequencies of vectors collected in each geographical area are summarized in Supplementary Table 2. *An. funestus* group was the most prevalent vector in all areas. Of the 272 mosquitoes collected by HLC, 3.3% (9/272) tested positive for *P. falciparum* sporozoites. Higher sporozoite rate was observed among the *An. funestus group* (7/9). The rate of indoor and outdoor biting estimated by HLC was 19.8 and 25.5 bites per person per night, respectively.”**

**Discussion section**

**“Lack of explicit molecular data for distinguishing sibling species and molecular forms within the *An. funestusgroup* introduces ambiguity into the interpretation of the results of the study.”**

Q3) The authors reported that PCR (polymerase chain reaction) was done on the mosquitoes yet I cannot find data in the paper reporting the outcome of the PCR. All data reports *An. gambiae s.l. a*nd *An. funestus group*.

**A3: We have addressed this comment as shown above.**

Q4) The paper explores changing mosquito behaviour with lowered effectiveness of nets but only used one month of vector collections two years after the clinical data was collected to test this link and the actual species present are not reported. I therefore find this a big stretch of the data. Vector density, composition and behaviour varies throughout the year and these collections were made for a short time. I therefore don’t think the data are sufficient to accept or reject the hypothesis. 

**A4: We have updated the clinical surveillance data to December 2016 and updated the manuscript accordingly. We have included the limitation of the one month vector collection in the discussion section as shown below.**

**Discussion section**

**“The size of our study limits power: with a sample size of 415, and the proportion of mosquitoes biting outdoors at 67% in low ITN effectiveness areas we therefore had >90% power to detect a reduction to 27% or lower in high ITN effectiveness areas. Our study was therefore powered to detect only a large difference in the proportion of vectors caught outdoors. However, we reasoned that reductions of ITN effectiveness to less than half of the previously documented efficacy of 50% would require a doubling of the proportion of mosquitoes feeding outdoors. Hence our study was powered to detect large variations in the frequency of outdoor biting. In addition, the accuracy of mosquito sampling data is limited as only one month of sampling was conducted in this study, we recommend sampling for a longer duration of time.”**

Q5) That being said the rest of the data is very useful and nicely presented. The data do demonstrate that there is substantial outdoor biting in June/July, and I should like to see the species composition in the area seeing as the authors report that the PCR was done. Outdoor biting may not increase malaria if the vectors doing the outdoor biting are not very competent for malaria.

**A5: We have addressed this comment as shown above.**

# Peer review: Referee response for version 1

**DOI:** 10.21956/wellcomeopenres.11943.r22171

**Published:** 2017-05-18

**Authors:** Gerry F Killeen

**Affiliations:** 1 Environmental Health and Ecological Sciences Thematic group, Ifakara Health Institute, Dar es Salaam, Tanzania

Apart from some unfortunately important exceptions, the data for this study are meticulously collected and analysed. However, many of the most important results are either over-interpreted or misinterpreted so these exceptions are substantive. In fact, the correct interpretation may well be almost the exact opposite of that presented here: That LLINs are consistently effective across a landscape where transmission is dominated by a vector that primarily attacks people indoors at night while they are asleep.

1. The biggest single problem with this paper is that the indoor and outdoor biting rate estimates come from stationary, fully exposed human volunteers exhibiting artificial experimental behaviours, without adjusting them for normal human behaviours that mean most of us are indoors asleep during the peak biting hours of nocturnal African malaria vector mosquitoes. This is an understandable and common mistake, but a very important one. Like *Anopheles funestus* in most locations across Africa, the 55-45 distribution of biting location preference for this population is essentially indiscriminate, so it is the behaviour of humans that determines where exposure actually occurs. So unless everyone in coastal Kenya sleeps half indoors and half outdoors throughout the night, simply comparing indoor versus outdoor HLCs is misrepresentative and greatly exaggerates the contribution of outdoor biting to transmission by this species. Once adjusted for human behaviour patterns, >90% of human biting exposure to this key vector species is consistently estimated to occur indoors in the absence of some protective measures at locations scattered all across Africa [1]. Unless human behaviour on the coast of Kenya is far more exophilic (everyone sleeps outdoors?) than all the other human populations we have data for, there is nothing in the data presented that is unusual or that convince me this vector population behaves differently from *Anopheles funestus* elsewhere. The logical conclusion of this paper (albeit with some additional data and analyses to support it) is that, unsurprisingly, there is little difference in the effectiveness of nets across landscapes dominated by the same vector that primarily encounters people indoors at night while they are asleep and can use a net.
2. The most important data clearly missing from the characterization of the study scenario are (a) spoorozoite rates (mentioned in the methods but not the results) and EIR estimates, to confirm that *Anopheles funestus* group mosquitoes are the most important vectors of malaria in this area, (b) quantitative estimates of where and when humans are exposed to these two major vector taxa (not species unless PCR data are added) that weight the biting estimates by surveys of human behaviour [2-5]. These are increasingly common calculations applied to data from all over the tropics [6-13], and vitally important to conduct before making any quantitative statements about proportional contributions of outdoor biting exposure.
3. There is no evidence of any “shift” in behaviours over time presented here, so the term “undermines” is unjustified and seems to create an argument that hasn’t been made. Most behaviours that enable residual malaria transmission despite LLIN use are pre-existing, although plastic, and often it’s just the vector population composition that shifts 14, so the term “limits” is more appropriate.
4. While indeed there is no evidence here that outdoor transmission contributes to ongoing transmission, there is also no evidence that it does not. Such outdoor fractions of transmission can only be expected to become epidemiologically detectable once larger quantities of indoor transmission (which I’m convinced is the case here as explained above) have been tackled. So the phraseology of conclusions needs to be tempered using words like “yet”, and explain how these currently minor fractions of transmission may emerge as important contributors to sustained endemicity once further progress has been made with indoor control [14,15].
5. In any case, LLINs clearly fall a long way short of being 100% efficacious with 22% personal protection estimated here, so there clearly are considerable limitations to this technology that need explanation. To get a better handle on whether outdoor exposure does contribute to residual transmission, in our experience it’s necessary to test as a function of individual human behavioural profiles weighted by activity patterns for the most dominant local vectors [13]. Indeed human behaviour is the primary driver of where and when exposure occurs [1] and is far more variable than the mosquito behaviours that matter within a single vector species [15].
6. In any case, for many of the surveyed locations, very few mosquitoes were caught (Supplementary Table 2) and CDC light traps catches indoors and outdoors are not comparable, so reporting these data as indicators of the degree of exophagy or endophagy is going too far and overstretching very little entomological data.
7. The fact that these are not differentiated to species (again, though this is mentioned in the methods but no results are presented) also means that areas with apparently different mosquito behaviours are probably areas that simply have different relative abundances of primary vector, secondary vector and non-vector species within the *Anopheles funestus* group and within the *Anopheles gambiae* complex. For example, greater outdoor feeding at dawn and dusk is a known characteristic of *Anopheles rivulorum* and *Anopheles parensis*, originally discovered in this region on the basis of their distinctive behaviours and much weaker vectorial capacities.
8. The term “species” is used very loosely and interchangeably with other taxonomic classification levels, resulting in some misleading over-interpretation. While *Anopheles gambiae sensu lato* is indeed a complex, *Anopheles funestus sensu lato *is a group (not a complex, as stated in the introduction) and neither can be described as a species, unless one is talking about unambiguously identified individual specimens of the nominate species, which are by far the most efficient species within each taxon.
9. All of these most important limitations seem to be missing from the paragraph opening with the sentence “Our study has some limitations”.
10. What is called “effectiveness” here refers only to the relatively minor personal protection effect of bednets, and does not capture any variations in community-level impact. All fine but please explain this study limitation clearly.
11. Correspondingly, doesn’t capture how big a change this transmission picture is relative to the same setting 10 to 15 years ago when nominate *Anopheles gambiae* were still quite abundant. The explanations about the relative abundance of vector taxa (not species) is accurate but rather static and lacking in long term context, demonstrating the much bigger overall impact on vector populations and endemicity. This is a pity when this contemporary study has been conducted in an area with so much historical entomological literature, so please enrich the narrative.
12. While I agree with the closing statement about enhancing entomological surveillance, in my experience many groups are under-interpreting or misinterpreting the data they already have, so perhaps that capacity limitation merits some emphasis as a priority.

I have read this submission. I believe that I have an appropriate level of expertise to confirm that it is of an acceptable scientific standard, however I have significant reservations, as outlined above.

## References

[1] HuhoBBriëtOSeyoumASikaalaCBayohNGimnigJOkumuFDialloDAbdullaSSmithTKilleenG: Consistently high estimates for the proportion of human exposure to malaria vector populations occurring indoors in rural Africa. *Int J Epidemiol*.2013;42(1) : 10.1093/ije/dys214 235-47 10.1093/ije/dys214 23396849PMC3600624

[2] KilleenGFKihondaJLyimoEOketchFRKotasMEMathengeESchellenbergJALengelerCSmithTADrakeleyCJ: Quantifying behavioural interactions between humans and mosquitoes: evaluating the protective efficacy of insecticidal nets against malaria transmission in rural Tanzania. *BMC Infect Dis*.2006;6: 10.1186/1471-2334-6-161 161 10.1186/1471-2334-6-161 17096840PMC1657018

[3] SeyoumASikaalaCHChandaJChinulaDNtamatungiroAJHawelaMMillerJMRussellTLBriëtOJKilleenGF: Human exposure to anopheline mosquitoes occurs primarily indoors, even for users of insecticide-treated nets in Luangwa Valley, South-east Zambia. *Parasit Vectors*.2012;5: 10.1186/1756-3305-5-101 101 10.1186/1756-3305-5-101 22647493PMC3432592

[4] KilleenGF: A second chance to tackle African malaria vector mosquitoes that avoid houses and don't take drugs. *Am J Trop Med Hyg*.2013;88(5) : 10.4269/ajtmh.13-0065 809-16 10.4269/ajtmh.13-0065 23589532PMC3752742

[5] KilleenGF: Characterizing, controlling and eliminating residual malaria transmission. *Malar J*.2014;13: 10.1186/1475-2875-13-330 330 10.1186/1475-2875-13-330 25149656PMC4159526

[6] BradleyJLinesJFuseiniGSchwabeCMontiFSlotmanMVargasDGarciaGHergottDKleinschmidtI: Outdoor biting by Anopheles mosquitoes on Bioko Island does not currently impact on malaria control. *Malar J*.2015;14: 10.1186/s12936-015-0679-2 170 10.1186/s12936-015-0679-2 25895674PMC4429929

[7] MoirouxNDamienGBEgrotMDjenontinAChandreFCorbelVKilleenGFPennetierC: Human exposure to early morning Anopheles funestus biting behavior and personal protection provided by long-lasting insecticidal nets. *PLoS One*.2014;9(8) : 10.1371/journal.pone.0104967 e104967 10.1371/journal.pone.0104967 25115830PMC4130624

[8] GeissbühlerYChakiPEmidiBGovellaNJShirimaRMayagayaVMtasiwaDMshindaHFillingerULindsaySWKannadyKde CastroMCTannerMKilleenGF: Interdependence of domestic malaria prevention measures and mosquito-human interactions in urban Dar es Salaam, Tanzania. *Malar J*.2007;6: 10.1186/1475-2875-6-126 126 10.1186/1475-2875-6-126 17880679PMC2039744

[9] GovellaNJOkumuFOKilleenGF: Insecticide-treated nets can reduce malaria transmission by mosquitoes which feed outdoors. *Am J Trop Med Hyg*.2010;82(3) : 10.4269/ajtmh.2010.09-0579 415-9 10.4269/ajtmh.2010.09-0579 20207866PMC2829902

[10] BugoroHCooperRDButafaCIro'ofaCMackenzieDOChenCCRussellTL: Bionomics of the malaria vector Anopheles farauti in Temotu Province, Solomon Islands: issues for malaria elimination. *Malar J*.2011;10: 10.1186/1475-2875-10-133 133 10.1186/1475-2875-10-133 21592366PMC3123245

[11] RussellTLGovellaNJAziziSDrakeleyCJKachurSPKilleenGF: Increased proportions of outdoor feeding among residual malaria vector populations following increased use of insecticide-treated nets in rural Tanzania. *Malar J*.2011;10: 10.1186/1475-2875-10-80 80 10.1186/1475-2875-10-80 21477321PMC3084176

[12] RussellTLBeebeNWBugoroHApairamoAChowWKCooperRDCollinsFHLoboNFBurkotTR: Frequent blood feeding enables insecticide-treated nets to reduce transmission by mosquitoes that bite predominately outdoors. *Malar J*.2016;15: 10.1186/s12936-016-1195-8 156 10.1186/s12936-016-1195-8 26969430PMC4788858

[13] MsellemuDNamangoHIMwakalingaVMNtamatungiroAJMlachaYMtemaZJKiwareSLoboNFMajambereSDongusSDrakeleyCJGovellaNJChakiPPKilleenGF: The epidemiology of residual Plasmodium falciparum malaria transmission and infection burden in an African city with high coverage of multiple vector control measures. *Malar J*.2016;15(1) : 10.1186/s12936-016-1340-4 288 10.1186/s12936-016-1340-4 27216734PMC4877954

[14] GovellaNJChakiPPKilleenGF: Entomological surveillance of behavioural resilience and resistance in residual malaria vector populations. *Malar J*.2013;12: 10.1186/1475-2875-12-124 124 10.1186/1475-2875-12-124 23577656PMC3637503

[15] KilleenGFMarshallJMKiwareSSSouthABTustingLSChakiPPGovellaNJ: Measuring, manipulating and exploiting behaviours of adult mosquitoes to optimise malaria vector control impact. *BMJ Global Health*.2017;2(2) :p.e000212 10.1136/bmjgh-2016-000212PMC544408528589023

# Response

**Published:** 2018-01-09

**Authors:** Alice Kamau

**Affiliations:** KEMRI-Wellcome Trust Research Programme, Kenya

We are grateful for this review and the helpful comments and suggestions that have been made. We have included a point-by-point response (in bold) to the issues raised.

Q1) The biggest single problem with this paper is that the indoor and outdoor biting rate estimates come from stationary, fully exposed human volunteers exhibiting artificial experimental behaviours, without adjusting them for normal human behaviours that mean most of us are indoors asleep during the peak biting hours of nocturnal African malaria vector mosquitoes. This is an understandable and common mistake, but a very important one. Like Anopheles funestus in most locations across Africa, the 55-45 distribution of biting location preference for this population is essentially indiscriminate, so it is the behaviour of humans that determines where exposure actually occurs. So unless everyone in coastal Kenya sleeps half indoors and half outdoors throughout the night, simply comparing indoor versus outdoor HLCs is misrepresentative and greatly exaggerates the contribution of outdoor biting to transmission by this species. Once adjusted for human behaviour patterns, >90% of human biting exposure to this key vector species is consistently estimated to occur indoors in the absence of some protective measures at locations scattered all across Africa [1]. Unless human behaviour on the coast of Kenya is far more exophilic (everyone sleeps outdoors?) than all the other human populations we have data for, there is nothing in the data presented that is unusual or that convince me this vector population behaves differently from Anopheles funestus elsewhere. The logical conclusion of this paper (albeit with some additional data and analyses to support it) is that, unsurprisingly, there is little difference in the effectiveness of nets across landscapes dominated by the same vector that primarily encounters people indoors at night while they are asleep and can use a net.

**A1:  We have made adjustments to the indoor and outdoor biting rate by human behavior as follows in the following sections:**

**Methods section**

**“To determine the human-mosquito contact, we administered questionnaires to 304 randomly selected households in the six selected areas between September and October 2016. We asked the household head time when each household member went to sleep and the time they woke up. Data on human behaviour was used to make adjustments to the indoor and outdoor biting rate.”**

**Statistical analysis section**

**“Questionnaire data about the time household members went to sleep and at what time they woke up were combined with human landing catches measurements of hourly rates for indoor and outdoor biting. We estimated the proportion of human exposure to mosquito bites occurring indoors (π _s_) by taking into consideration the movement pattern of people using the following method [1]: by weighting the mean indoor and outdoor biting rates throughout the night by the proportion of humans reporting to have gone to sleep at each hour of the night as follows;**

**π _s _= Σ ( B _*i,t*_* *S *_t _) / *Σ ( B _*i,t*_* *S *_t  _+ *B *_o,t_ ( *1 - S *_t _*))**

**(1)**

**Where:**

**= an estimate of human exposure to bites which occurs when residents are both indoors and sleeping**

**S _t_ = the proportion of humans indoors reporting to have gone to sleep at each hour of the night (t)**

**B _i,t_ = mean indoor biting rate at each hour of the night (t)**

**B _o,t_ = mean outdoor biting rates at each hour of the night (t)**

**(1-S _t_) = proportion of humans not yet asleep at each hour of the night.”**

**Result section**

**“Seventy three percent of children <5 years were reported to be asleep between 6 pm and 9 pm, these rose monotonically over the course of the night reaching 100% by 10 pm (Table 4 & Figure 6). Children aged between 6-14 years spent more time awake, only 45% were asleep before 9 pm (Figure 6 & Supplementary Table 3). Human landing catches are not sufficient in themselves to survey normal human exposure to mosquito bite. The timing of human activity and sleeping behaviour in particular modulates the effect of human-mosquito contact and the effectiveness of ITN. We quantified the interaction between mosquitoes and humans to evaluate whether outdoor vector biting is a potential explanation for the variation in ITN effectiveness. The peak biting activity for each mosquito vector is illustrated in Figure 7. Clearly higher indoor biting activity was observed for the An. funestus group. The overall propensity to feed at times when most people are indoor was high (Figure 8): the vast majority of the Anopheles mosquitoes were caught at times when most people are indoors (Figure 7). Estimates for the proportion of human-mosquito contact between the first and last hour when most humans were indoors was consistently high, ranging from 0.83 to 1.00. Therefore, the estimated proportion of exposure to Anopheles mosquito bites that occurred indoor was high.”**

**Discussion section**

**It is possible that a higher proportion of mosquitoes caught outdoors represents a behavioral response to unsuccessful feeding attempts made indoors during the night, and therefore it may simply be a marker of successful ITN use. This avoidance behavior may exert a cost on the vector, and so ITNs may in fact still be protective in areas where outdoor biting is observed, as has been suggested previously [2]. Furthermore, outdoor biting exposure and the probability of successful feeding outdoors cannot be directly inferred from the human landing catches, since the landing catches are not in themselves sufficient to survey pattern of normal human exposure to mosquito bite. Once adjusted for human behaviour, most human-vector interaction in this study occurred indoors (Figure 8 & Supplementary Table 3). Outdoor biting is currently not a major factor influencing residual malaria transmission since 95% of the population are indoors at the peak biting period for malaria vector mosquitoes. Human behaviour is the primary driver of when and where exposure occurs and is far more variable than the mosquito behaviour that matter within a single vector species [3].**

Q2) The most important data clearly missing from the characterization of the study scenario are (a) spoorozoite rates (mentioned in the methods but not the results) and EIR estimates, to confirm that Anopheles funestus group mosquitoes are the most important vectors of malaria in this area, (b) quantitative estimates of where and when humans are exposed to these two major vector taxa (not species unless PCR data are added) that weight the biting estimates by surveys of human behaviour [2-5]. These are increasingly common calculations applied to data from all over the tropics [6-13], and vitally important to conduct before making any quantitative statements about proportional contributions of outdoor biting exposure.

**A2:** **We have included data obtained from the ELISA-CSP and molecular analysis.** **We have** **also** **added data on sporozoite rate as shown below in the result section.**

**Result section**

**“Over 26 nights, 415 female *Anopheles* mosquitoes were collected by both methods (i.e. 272 by HLC and 143 by CDC-LT), representing a mean of 16 mosquitoes per night. 66% of mosquitoes were collected using HLC. Of the 415 mosquitoes morphologically identified, 311 (75%) were *An. funestus group*, 84 (20%) were *An. gambiae s.l.* and 20 (5%) were other Anopheles i.e. *An. protoriensis, An. coustani, An. moucheti and An. squamosus* (Table 2). The An. funestus group was significantly greater than An. gambiae s.l (p<0.001). Out of the 84 amplified samples of *An. gambiae s.l*., 68 (81%) were *An. Arabiensis* and 16 (19%) were *An. gambiae s.s.* The proportion of *Anopheles* mosquitoes caught outdoors (60%; 95% CI: 55%, 65%) was significantly greater than the proportion caught indoors (p<0.001). There were more *Anopheles* mosquitoes collected outdoors in all geographical areas except area 6, where most of the mosquitoes were collected indoor (Table 2). The frequencies of vectors collected in each geographical area are summarized in Supplementary Table 2. *An. funestus* group was the most prevalent vector in all areas. Of the 272 mosquitoes collected by HLC, 3.3% (9/272) tested positive for *P. falciparum* sporozoites. Higher sporozoite rate was observed among the *An. funestus group* (7/9). The rate of indoor and outdoor biting estimated by HLC was 19.8 and 25.5 bites per person per night, respectively.”**

Q3) There is no evidence of any “shift” in behaviours over time presented here, so the term “undermines” is unjustified and seems to create an argument that hasn’t been made. Most behaviours that enable residual malaria transmission despite LLIN use are pre-existing, although plastic, and often it’s just the vector population composition that shifts 14, so the term “limits” is more appropriate.

**A3: We have revised as proposed above in the abstract section.**

**Conclusion**

**“Our data therefore do not support the hypothesis that outdoor biting limits the effectiveness of ITNs in our study area.”**

Q4) While indeed there is no evidence here that outdoor transmission contributes to ongoing transmission, there is also no evidence that it does not. Such outdoor fractions of transmission can only be expected to become epidemiologically detectable once larger quantities of indoor transmission (which I’m convinced is the case here as explained above) have been tackled. So the phraseology of conclusions needs to be tempered using words like “yet”, and explain how these currently minor fractions of transmission may emerge as important contributors to sustained endemicity once further progress has been made with indoor control [14,15].

**A4: We have revised as proposed above in the discussion section.**

**Discussion section**

**“In summary, our data do not support the hypothesis that outdoor biting limits the effectiveness of ITNs in our study area. The outdoor biting observed may therefore have been the result of high levels of ITN use leading to unsuccessful attempts at indoor feeding. However, it remains possible that continued selection pressures might lead to the emergence of populations of mosquitoes that are better adapted to outdoor feeding in the future. Outdoor feeding is becoming more common in parts of Africa [4] and may represent evolutionary change in some areas, with a potential to undermine ITN effectiveness. The outdoor fractions of transmission can be expected to be epidemiologically detectable once indoor transmission has been tackled. Therefore, malaria control programs require monitoring to assess the impact of ITNs on vector populations and vector behavioral change as well as monitoring ITN effectiveness as vectors evolve [5-9]. Continuous monitoring of vector bionomics, and malaria transmission dynamics are essential for predicting disease outbreaks and guiding vector control in the region. Furthermore, capacity needs to be built in interpreting and applying these data to malaria control policy.”**

Q5) In any case, LLINs clearly fall a long way short of being 100% efficacious with 22% personal protection estimated here, so there clearly are considerable limitations to this technology that need explanation. To get a better handle on whether outdoor exposure does contribute to residual transmission, in our experience it’s necessary to test as a function of individual human behavioural profiles weighted by activity patterns for the most dominant local vectors [13]. Indeed human behaviour is the primary driver of where and when exposure occurs [1] and is far more variable than the mosquito behaviours that matter within a single vector species [15].

**A5:  We have made adjustments to the indoor and outdoor biting rate by human behavior as shown above, and accordingly revised the discussion as above.**

Q6) In any case, for many of the surveyed locations, very few mosquitoes were caught (Supplementary Table 2) and CDC light traps catches indoors and outdoors are not comparable, so reporting these data as indicators of the degree of exophagy or endophagy is going too far and overstretching very little entomological data.

**A6: We have made revision in the result and discussion section as shown above.**

Q7) The fact that these are not differentiated to species (again, though this is mentioned in the methods but no results are presented) also means that areas with apparently different mosquito behaviours are probably areas that simply have different relative abundances of primary vector, secondary vector and non-vector species within the Anopheles funestus group and within the Anopheles gambiae complex. For example, greater outdoor feeding at dawn and dusk is a known characteristic of Anopheles rivulorum and Anopheles parensis, originally discovered in this region on the basis of their distinctive behaviours and much weaker vectorial capacities.

**A7: We have addressed the above comment as follows:**

**We have included Figure 7 which illustrates hourly biting pattern of Anopheles mosquitoes occurring both indoors (solid lines) and outdoors (dashed lines). The grey area represents the proportion of the children <5 years asleep at each hour of the night.**

**We do not have molecular data for the *An. funestus group*. We have included this as a limitation in the discussion section as shown below.**

**Discussion section**

**“Lack of explicit molecular data for distinguishing sibling species and molecular forms within the *An. funestus group* introduces ambiguity into the interpretation of the results of the study.”**

Q8) The term “species” is used very loosely and interchangeably with other taxonomic classification levels, resulting in some misleading over-interpretation. While Anopheles gambiae sensu lato is indeed a complex, Anopheles funestus sensu lato is a group (not a complex, as stated in the introduction) and neither can be described as a species, unless one is talking about unambiguously identified individual specimens of the nominate species, which are by far the most efficient species within each taxon.

**A8: We have made revisions accordingly.**

Q9) All of these most important limitations seem to be missing from the paragraph opening with the sentence “Our study has some limitations”.

**A9: We have updated the manuscript with the adjustments to the indoor and outdoor biting rate by human behaviour as shown above. We have also updated the limitations of our study as shown under the discussion section.**

**Discussion section**

**“Our study has a number of limitations. Data on ITN use may have been incorrectly reported, as we did not require each resident to be present during the survey. We attempted to minimize this by instructing data collecting teams to interview only residents of the same homestead regarding ITN ownership and usage. There may have been some misclassification as we did not ascertain ITN use during hospital presentation but instead used the yearly ITN data collected by the annual survey. The results may also be confounded by other unmeasured factors (e.g., variation in the quality and type of ITN, urbanization, socio-economic status and mother’s education). It is likely that we underestimated the protection afforded by the use of high-quality ITN because we included all ITNs, regardless of quality, physical integrity or bioefficacy of the insecticidal compounds. The vast majority of ITNs in the area are long-lasting insecticidal nets, hence we do not expect substantial variation in insecticidal efficacy. The accuracy of the mosquito survey is limited by the practical challenges of maintaining consistently sensitive human landing catches throughout the night. Lack of explicit molecular data for distinguishing sibling species and molecular forms within the An. funestus group introduces ambiguity into the interpretation of the results of the study. In this study, we examined variations in the personal protection afforded by ITNs and did not examine variation in community level effect. The size of our study limits power: with a sample size of 415, and the proportion of mosquitoes biting outdoors at 67% in low ITN effectiveness areas we therefore had >90% power to detect a reduction to 27% or lower in high ITN effectiveness areas. Our study was therefore powered to detect only a large difference in the proportion of vectors caught outdoors. However, we reasoned that reductions of ITN effectiveness to less than half of the previously documented efficacy of 50% would require a doubling of the proportion of mosquitoes feeding outdoors.  Hence our study was powered to detect large variations in the frequency of outdoor biting. In addition, the accuracy of mosquito sampling data is limited as only one month of sampling was conducted in this study, we recommend sampling for a longer duration of time.”** 

Q10) What is called “effectiveness” here refers only to the relatively minor personal protection effect of bednets, and does not capture any variations in community-level impact. All fine but please explain this study limitation clearly.

**A10: We have made revision in the discussion section indicating this limitation as follows.**

**Discussion section**

**In this study, we examined variations in the personal protection afforded by ITNs and did not examine variation in community level effect.**

Q11) Correspondingly, doesn’t capture how big a change this transmission picture is relative to the same setting 10 to 15 years ago when nominate Anopheles gambiae were still quite abundant. The explanations about the relative abundance of vector taxa (not species) is accurate but rather static and lacking in long term context, demonstrating the much bigger overall impact on vector populations and endemicity. This is a pity when this contemporary study has been conducted in an area with so much historical entomological literature, so please enrich the narrative.

**A11: We have added points as follows in the discussion section;**

**“Malaria transmission has reduced dramatically over the last 15 years in Kilifi, evidenced by falling rates of clinical malaria cases in hospital [10, 11] in the community [12] and falling community prevalence of asymptomatic infection [13]. A recent resurgence has been noted with increasing cases among older children, and increasing prevalence of infection more widely around the coast [14]. The reductions have been temporally associated with marked reductions in the prevalence of the abundance of vectors [15] and with a pronounced shift away from *Anopheles gambiae s.s*, which was previously the dominant vector, and a shift away from *Anopheles arabiensis*.”**

Q12) While I agree with the closing statement about enhancing entomological surveillance, in my experience many groups are under-interpreting or misinterpreting the data they already have, so perhaps that capacity limitation merits some emphasis as a priority.

**A12: We have added a statement in the summary section as shown below.**

**“Furthermore, capacity needs to be built in interpreting and applying these data to malaria control policy.”**

**References**

1.              Seyoum A, Sikaala CH, Chanda J, Chinula D, Ntamatungiro AJ, Hawela M, Miller JM, Russell TL, Briet OJ, Killeen GF: **Human exposure to anopheline mosquitoes occurs primarily indoors, even for users of insecticide-treated nets in Luangwa Valley, South-east Zambia.** *Parasit Vectors *2012, **5:**101.

2.              Govella NJ, Okumu FO, Killeen GF: **Insecticide-treated nets can reduce malaria transmission by mosquitoes which feed outdoors.** *Am J Trop Med Hyg *2010, **82:**415-419.

3.              Huho B, Briet O, Seyoum A, Sikaala C, Bayoh N, Gimnig J, Okumu F, Diallo D, Abdulla S, Smith T, Killeen G: **Consistently high estimates for the proportion of human exposure to malaria vector populations occurring indoors in rural Africa.** *Int J Epidemiol *2013, **42:**235-247.

4.              Githeko AK, Adungo NI, Karanja DM, Hawley WA, Vulule JM, Seroney IK, Ofulla AV, Atieli FK, Ondijo SO, Genga IO, et al: **Some observations on the biting behavior of Anopheles gambiae s.s., Anopheles arabiensis, and Anopheles funestus and their implications for malaria control.** *Exp Parasitol *1996, **82:**306-315.

5.              Mutuku FM, King CH, Mungai P, Mbogo C, Mwangangi J, Muchiri EM, Walker ED, Kitron U: **Impact of insecticide-treated bed nets on malaria transmission indices on the south coast of Kenya.** *Malar J *2011, **10:**356.

6.              Bayoh MN, Mathias DK, Odiere MR, Mutuku FM, Kamau L, Gimnig JE, Vulule JM, Hawley WA, Hamel MJ, Walker ED: **Anopheles gambiae: historical population decline associated with regional distribution of insecticide-treated bed nets in western Nyanza Province, Kenya.** *Malar J *2010, **9:**62.

7.              Gimnig JE, Kolczak MS, Hightower AW, Vulule JM, Schoute E, Kamau L, Phillips-Howard PA, ter Kuile FO, Nahlen BL, Hawley WA: **Effect of permethrin-treated bed nets on the spatial distribution of malaria vectors in western Kenya.** *Am J Trop Med Hyg *2003, **68:**115-120.

8.              Russell TL, Govella NJ, Azizi S, Drakeley CJ, Kachur SP, Killeen GF: **Increased proportions of outdoor feeding among residual malaria vector populations following increased use of insecticide-treated nets in rural Tanzania.** *Malar J *2011, **10:**80.

9.              Killeen GF, Kihonda J, Lyimo E, Oketch FR, Kotas ME, Mathenge E, Schellenberg JA, Lengeler C, Smith TA, Drakeley CJ: **Quantifying behavioural interactions between humans and mosquitoes: evaluating the protective efficacy of insecticidal nets against malaria transmission in rural Tanzania.** *BMC Infect Dis *2006, **6:**161.

10.           Mogeni P, Williams TN, Fegan G, Nyundo C, Bauni E, Mwai K, Omedo I, Njuguna P, Newton CR, Osier F, et al: **Age, Spatial, and Temporal Variations in Hospital Admissions with Malaria in Kilifi County, Kenya: A 25-Year Longitudinal Observational Study.** *PLoS Med *2016, **13:**e1002047.

11.           O'Meara WP, Bejon P, Mwangi TW, Okiro EA, Peshu N, Snow RW, Newton CR, Marsh K: **Effect of a fall in malaria transmission on morbidity and mortality in Kilifi, Kenya.** *Lancet *2008, **372:**1555-1562.

12.           Mwangi TW, Ross A, Snow RW, Marsh K: **Case definitions of clinical malaria under different transmission conditions in Kilifi District, Kenya.** *J Infect Dis *2005, **191:**1932-1939.

13.           Mogeni P, Williams TN, Omedo I, Kimani D, Ngoi JM, Mwacharo J, Morter R, Nyundo C, Wambua J, Nyangweso G: **Detecting Malaria Hotspots: a comparison between RDT, Microscopy and Polymerase Chain Reaction.** *The Journal of Infectious Diseases *2017.

14.           Snow RW, Kibuchi E, Karuri SW, Sang G, Gitonga CW, Mwandawiro C, Bejon P, Noor AM: **Changing Malaria Prevalence on the Kenyan Coast since 1974: Climate, Drugs and Vector Control.** *PLoS One *2015, **10:**e0128792.

15.           Mwangangi JM, Mbogo CM, Orindi BO, Muturi EJ, Midega JT, Nzovu J, Gatakaa H, Githure J, Borgemeister C, Keating J, Beier JC: **Shifts in malaria vector species composition and transmission dynamics along the Kenyan coast over the past 20 years.** *Malar J *2013, **12:**13.

# Peer review: Referee response for version 1

**DOI:** 10.21956/wellcomeopenres.11943.r22390

**Published:** 2017-05-02

**Authors:** Seynabou Sougoufara

**Affiliations:** 1 Département de Biologie Animale, Faculté des Sciences et Techniques, Université Cheikh Anta Diop, Dakar Sénégal, France; 2 Unité de Recherche sur les Maladies Infectieuses et Tropicales Emergentes, UM63, CNRS 7278, IRD 198, INSERM 1095, IHU - Méditerranée Infection, Marseille, France

The authors present here the study on the variation of the effectiveness of insecticide-treated bed nets against malaria and the outdoor biting by vectors in Kilifi, Kenya.

 The manuscript reported the geographical heterogeneity of malaria prevalence according several parameters mainly including the ITN effectiveness and the feeding behaviour of *Anopheles* vectors. The design and method of the study are well presented in the section “Methods” as well as the statistical analysis. Clinical surveillance was analyzed in the study between January 2009 and December 2014 that covers a long period. Thus it will be interesting if authors add in their explanatory factors the dry and wet season. It will be also important to explain the discrepancy between the date of clinical surveillance data collection (January 2009 and December 2014) and the mosquito collection (July and August 2016). We have any informations if the level of ITN use varied or is the same during both periods.

 Additionally the main part of the subject underlines the effectiveness of the ITNs. However, authors should describe at first that the effectiveness of ITNs is monitoring taking into account the physical integrity of nets, bioefficacity and the insecticidal compounds even though they focused more their study on feeding place and malaria prevalence. It will be also more appropriate if authors interpreted their result according to level of ITNs use according to areas and discuss though their outcomes the effectiveness of ITN. For instance in the abstract the expression of “ *high and low effectiveness*” in the part of method is a hasty affirmation.

 In the section of “Results” I think that the Supplementary Table 1 has to be presented in the main manuscript as it present malaria prevalence according to area and the level of ITNs use. Moreover the presentation of results must be more detailed and the effect of each risk factors cited in the part of “Statistical analysis” must be presented. I don’t understand why authors said “ *ITN use was consistently >50% in all geographical areas”, *meaning that here we have no information about the difference of level use between areas. The authors have summarized too much the description of the results in this part.

 Authors presented in the part “Mosquito processing”  laboratory works such ELISA-CSP and molecular analysis, however the results of these analysis have not been presented in this study.

 Regarding the result on vector abundance, authors have to present the results according to absolute densities and less on the proportion of species in the place of mosquito collection.

 The relevance of the study will be more remarkable if authors greatly discuss in deep their outcomes by comparing with other studies. Additionally, the review of the literature has to be strengthened, “33 off the 43 references are more than 5 years old and some newer papers are missing.

I have read this submission. I believe that I have an appropriate level of expertise to confirm that it is of an acceptable scientific standard, however I have significant reservations, as outlined above.

# Response

**Published:** 2018-01-06

**Authors:** Alice Kamau

**Affiliations:** KEMRI-Wellcome Trust Research Programme, Kenya

We are grateful for this review and the helpful comments and suggestions that have been made. We have included a point-by-point response (in bold) to the issues raised.

Q1) The manuscript reported the geographical heterogeneity of malaria prevalence according several parameters mainly including the ITN effectiveness and the feeding behaviour of Anopheles vectors. The design and method of the study are well presented in the section “Methods” as well as the statistical analysis. Clinical surveillance was analyzed in the study between January 2009 and December 2014 that covers a long period. Thus it will be interesting if authors add in their explanatory factors the dry and wet season. It will be also important to explain the discrepancy between the date of clinical surveillance data collection (January 2009 and December 2014) and the mosquito collection (July and August 2016). We have any information if the level of ITN use varied or is the same during both periods.

**A1:** **We have made revision to address all the questions above as follows:**

**We have updated the clinical surveillance data to December 2016 and updated the manuscript accordingly.** **We have also included season as a covariate in both univariable and multivariable analysis, Supplementary Table 1.**

**Changes are as follows;**

**Method section**

**“The clinical surveillance study was conducted between January 2009 and December 2016 within a 6km radius of Pingilikani dispensary in Kilifi County on the Kenyan Coast (Figure 1): within the Kilifi Health and Demographic Surveillance System (KHDSS).”**

**Statistical analysis section**

**“The outcome of interest was presence of malaria by microscopy on presentation to the dispensary. The potential risk factors included: ITN use, age of the child, year of presentation to the dispensary, season (the wet season comprised of April, May, June, October and November) and the geographical area, as defined by the 2.5x2.5 km regular polygons.”**

Q2) Additionally the main part of the subject underlines the effectiveness of the ITNs. However, authors should describe at first that the effectiveness of ITNs is monitoring taking into account the physical integrity of nets, bioefficacity and the insecticidal compounds even though they focused more their study on feeding place and malaria prevalence. It will be also more appropriate if authors interpreted their result according to level of ITNs use according to areas and discuss though their outcomes the effectiveness of ITN. For instance, in the abstract the expression of “high and low effectiveness” in the part of method is a hasty affirmation.

**A2: We have included the above comment as a limitation to our study as we did not have data on the physical integrity of nets or the bioefficacy of the insecticidal compounds. We’ve also revised the abstract session.** **We’ve revised the abstract session to indicate “varying ITN effectiveness” rather than “high and low ITN effectiveness”**

**Abstract section**

**“We linked homestead level geospatial data to clinical surveillance data at a primary healthcare facility in Kilifi County in order to map geographical heterogeneity in ITN effectiveness and observed vector feeding behaviour using landing catches and CDC light traps in six selected areas of varying ITN effectiveness.”**

**Discussion section**

**“It is likely that we underestimated the protection afforded by the use of high-quality ITN because we included all ITNs, regardless of quality, physical integrity or bioefficacy of the insecticidal compounds.”**

Q3) In the section of “Results” I think that the Supplementary Table 1 has to be presented in the main manuscript as it present malaria prevalence according to area and the level of ITNs use. Moreover, the presentation of results must be more detailed and the effect of each risk factors cited in the part of “Statistical analysis” must be presented. I don’t understand why authors said “ITN use was consistently >50% in all geographical areas”, meaning that here we have no information about the difference of level use between areas. The authors have summarized too much the description of the results in this part. Authors presented in the part “Mosquito processing” laboratory works such ELISA-CSP and molecular analysis, however the results of these analysis have not been presented in this study. Regarding the result on vector abundance, authors have to present the results according to absolute densities and less on the proportion of species in the place of mosquito collection.

**A3:** **We have made revision to address all the questions above as follows:**

**We have included two tables: (i) a** **descriptive table in the main text that indicates the prevalence of malaria and ITN use; and (ii) a table showing the full result of the univariable and multivariable analysis. We have included data obtained from the ELISA-CSP and molecular analysis. We’ve made revisions as shown below.**

**Result section**

**We’ve have included a descriptive table in the main text that indicates the prevalence of malaria and ITN use i.e. Table 1.**

**The full result of the univariable and multivariable analysis are shown in the Supplementary Table 1.**

**“Over 26 nights, 415 female *Anopheles* mosquitoes were collected by both methods (i.e. 272 by HLC and 143 by CDC-LT), representing a mean of 16 mosquitoes per night. 66% of mosquitoes were collected using HLC. Of the 415 mosquitoes morphologically identified, 311 (75%) were *An. funestus group*, 84 (20%) were *An. gambiae s.l.* and 20 (5%) were other Anopheles i.e. *An. protoriensis, An. coustani, An. moucheti and An. squamosus* (Table 2). The An. funestus group was significantly greater than An. gambiae s.l (p<0.001). Out of the 84 amplified samples of *An. gambiae s.l*., 68 (81%) were *An. Arabiensis* and 16 (19%) were *An. gambiae s.s.* The proportion of *Anopheles* mosquitoes caught outdoors (60%; 95% CI: 55%, 65%) was significantly greater than the proportion caught indoors (p<0.001). There were more *Anopheles* mosquitoes collected outdoors in all geographical areas except area 6, where most of the mosquitoes were collected indoor (Table 2). The frequencies of vectors collected in each geographical area are summarized in Supplementary Table 2. *An. funestus* group was the most prevalent vector in all areas. Of the 272 mosquitoes collected by HLC, 3.3% (9/272) tested positive for *P. falciparum* sporozoites. Higher sporozoite rate was observed among the *An. funestus group* (7/9). The rate of indoor and outdoor biting estimated by HLC was 19.8 and 25.5 bites per person per night, respectively.”**

Q4) The relevance of the study will be more remarkable if authors greatly discuss in deep their outcomes by comparing with other studies. Additionally, the review of the literature has to be strengthened, “33 off the 43 references are more than 5 years old and some newer papers are missing.

**A4: We have added 10 more recent reference as shown under the reference section. We have discussed our outcomes by comparing with other studies under the discussion section**

**Reference section**

1. Bradley J, Lines J, Fuseini G, Schwabe C, Monti F, Slotman M, Vargas D, Garcia G, Hergott D, Kleinschmidt I: **Outdoor biting by Anopheles mosquitoes on Bioko Island does not currently impact on malaria control.** Malar J 2015, **14:**170.
2. Moiroux N, Damien GB, Egrot M, Djenontin A, Chandre F, Corbel V, Killeen GF, Pennetier C: **Human exposure to early morning Anopheles funestus biting behavior and personal protection provided by long-lasting insecticidal nets.** PLoS One 2014, **9:**e104967.
3. Killeen GF, Marshall JM, Kiware SS, South AB, Tusting LS, Chaki PP, Govella NJ: **Measuring, manipulating and exploiting behaviours of adult mosquitoes to optimise malaria vector control impact.** BMJ Glob Health 2017, **2:**e000212. 
4. Kamau A, Nyaga V, Bauni E, Tsofa B, Noor AM, Bejon P, Scott JAG, Hammitt LL: **Trends in bednet ownership and usage, and the effect of bednets on malaria hospitalization in the Kilifi Health and Demographic Surveillance System (KHDSS): 2008-2015.** BMC Infect Dis 2017, **17:**720.
5. Royston P, Sauerbrei W: **Building multivariable regression models with continuous covariates in clinical epidemiology--with an emphasis on fractional polynomials.** Methods Inf Med 2005, **44:**561-571.
6. Sauerbrei W, Meier-Hirmer C, Benner A, Royston P: **Multivariable regression model building by using fractional polynomials: description of SAS, STATA and R programs.** Computational Statistics & Data Analysis 2006, **50:**3464-3485.
7. Kezdi G: **Robust Standard Error Estimation in Fixed-Effects Panel Models.** 2003.
8. Seyoum A, Sikaala CH, Chanda J, Chinula D, Ntamatungiro AJ, Hawela M, Miller JM, Russell TL, Briet OJ, Killeen GF: **Human exposure to anopheline mosquitoes occurs primarily indoors, even for users of insecticide-treated nets in Luangwa Valley, South-east Zambia.** Parasit Vectors 2012, **5:**101.
9. Lengeler C: **Insecticide-treated bed nets and curtains for preventing malaria.** Cochrane Database Syst Rev 2004 **:**CD000363.
10. Moiroux N, Gomez MB, Pennetier C, Elanga E, Djenontin A, Chandre F, Djegbe I, Guis H, Corbel V: **Changes in Anopheles funestus biting behavior following universal coverage of long-lasting insecticidal nets in Benin.** J Infect Dis 2012, **206:**1622-1629.
11. Mwangi TW, Ross A, Snow RW, Marsh K: **Case definitions of clinical malaria under different transmission conditions in Kilifi District, Kenya.** J Infect Dis 2005, **191:**1932-1939.
12. Mogeni P, Williams TN, Omedo I, Kimani D, Ngoi JM, Mwacharo J, Morter R, Nyundo C, Wambua J, Nyangweso G: **Detecting Malaria Hotspots: a comparison between RDT, Microscopy and Polymerase Chain Reaction.** The Journal of Infectious Diseases 2017.
13. Kapesa A, Kweka EJ, Atieli H, Kamugisha E, Zhou G, Githeko AK, Yan G: **Why some sites are responding better to anti-malarial interventions? A case study from western Kenya.** Malar J 2017, **16:**498.
14. Yohannes M, Boelee E: **Early biting rhythm in the Afro-tropical vector of malaria, Anopheles arabiensis, and challenges for its control in Ethiopia.** Med Vet Entomol 2012, **26:**103-105
15. Huho B, Briet O, Seyoum A, Sikaala C, Bayoh N, Gimnig J, Okumu F, Diallo D, Abdulla S, Smith T, Killeen G: **Consistently high estimates for the proportion of human exposure to malaria vector populations occurring indoors in rural Africa.** Int J Epidemiol 2013, **42:**235-247.

# Peer review: Referee response for version 1

**DOI:** 10.21956/wellcomeopenres.11943.r22156

**Published:** 2017-04-25

**Authors:** Heiko Becher

**Affiliations:** 1 Institute of Public Health, University of Heidelberg, Heidelberg, Germany

This report refers to statistical methods. Other relevant issues (“…  and does it cite the current literature?”) are not considered.

 The authors investigate the relation between ITN use and malaria prevalence, and secondly spatial variation in the effectiveness of ITN.

 Overall, the methods are too briefly described and make a thorough evaluation difficult. Some remarks may help to update the manuscript.

  

- The authors collected data from 20827 visits of 4992 children, i.e. about 5 visits for each child on average, from 21 areas. For each visit, parasitemia was assessed. The probability of parasitemia was modeled with a logistic regression model with ITN use, age, year and area as covariables, plus interaction terms. To account for correlated observations, a robust estimate of the standard errors was employed, although the exact method used is not given (reference should be provided). I wonder why season (rainy / dry) was not considered. The full result of the model is not given, and I wonder whether the large number of interaction terms in the model gave in a meaningful result. The Supplementary Table 1 gives the ORs for ITN use by area which is difficult to follow since (i) the numbering of the areas does not give information on spatial distribution (ii) it is not easy to see from the table whether malaria prevalence and ITN use differs between areas (iii) the effect of the other covariables is unknown (is there some confounding? What is the effect of age? Was a full fractional polynomial procedure used?).
- The Kulldorf statistic was used, if I understand correctly, to identify clusters of high or low ITN effectiveness without taking  malaria prevalence and ITN use into account. Is that true? This seems not correct to me but maybe I misunderstood the procedure.
- The proportion of vectors biting outdoors was compared for the areas. This would mean ignoring the absolute biting frequency which differs largely between areas.

Overall, the authors have carefully interpreted the results.

I have read this submission. I believe that I have an appropriate level of expertise to confirm that it is of an acceptable scientific standard, however I have significant reservations, as outlined above.

# Response

**Published:** 2018-01-06

**Authors:** Alice Kamau

**Affiliations:** KEMRI-Wellcome Trust Research Programme, Kenya

We are grateful for this review and the helpful comments and suggestions that have been made. We have included a point-by-point response (in bold) to the issues raised.

Q1) The authors collected data from 20827 visits of 4992 children, i.e. about 5 visits for each child on average, from 21 areas. For each visit, parasitemia was assessed. The probability of parasitemia was modeled with a logistic regression model with ITN use, age, year and area as covariables, plus interaction terms. To account for correlated observations, a robust estimate of the standard errors was employed, although the exact method used is not given (reference should be provided). I wonder why season (rainy / dry) was not considered. The full result of the model is not given, and I wonder whether the large number of interaction terms in the model gave in a meaningful result. The Supplementary Table 1 gives the ORs for ITN use by area which is difficult to follow since (i) the numbering of the areas does not give information on spatial distribution (ii) it is not easy to see from the table whether malaria prevalence and ITN use differs between areas (iii) the effect of the other covariables is unknown (is there some confounding? What is the effect of age? Was a full fractional polynomial procedure used?).

**A1:** **We have made revisions to address the questions raised above as follows:**

**Statistical analysis section**

**Wet vs. dry season was included as a covariate. We have included a reference for the multiple fractional polynomial transformation procedure [1,2]. We used the “mfp” command in STATA to assess the non-linear effect of age. We have also included a reference, which indicates what method was used for the robust standard error [3].**

**“The outcome of interest was presence of malaria by microscopy on presentation to the dispensary. The potential risk factors included: ITN use, age of the child, year of presentation to the dispensary, season (the wet season comprised of April, May, June, October and November) and the geographical area, as defined by the 2.5x2.5 km regular polygons.**

**To assess the non-linear effect of age in the regression models, multiple fractional polynomial transformation was used** **[1]** **. A list of fractional polynomial (FP) powers (–2, –1, –0.5, 0, 0.5, 1, 2, 3) were investigated for inclusion in the model using an algorithm that combines a backward elimination procedure with a search for an FP function that best predicts the outcome variable as previously described [2].**

**Given that the hospital malaria episodes were clustered within patients, we allowed for clustering by using a logistic regression model with robust standard errors [3].**

**Result section**

**We’ve have included a descriptive table in the main text indicating the prevalence of malaria and ITN use i.e. Table 1. We have included season as a covariate in both univariable and multivariable analysis, Supplementary Table 1. The full result of the univariable and multivariable analysis are shown in the Supplementary Table 1.** **We found the interaction terms to be significant and therefore retained them in the model.**

Q2) The Kulldorf statistic was used, if I understand correctly, to identify clusters of high or low ITN effectiveness without taking malaria prevalence and ITN use into account. Is that true? This seems not correct to me but maybe I misunderstood the procedure.

**A2: To compute the ITN effectiveness [i.e. (1 – OR) x 100] for each individual homestead, the outcome of interest was presence of malaria and the predictor was ITN use. We have included a description** **of SaTScan in the statistical analysis section as shown below:**

**Statistical analysis section**

**“SaTScan software (version 9.4; https://www.satscan.org/), a spatial scan statistic developed by Kulldorf[4], was used to detect potential spatial variations of ITN effectiveness (without smoothing) by identifying statistically significant geographical clustering of ITN effectiveness using the normal model. The space-time parameter of the spatial scan statistic places a cylindrical window on the coordinates grid for the locations studied and moves the center of the cylinder base over the grid so that the sets of geographic units covered by the window are constantly changing. Whenever the cylindrical window includes a new event, SaTScan calculates a likelihood function to test for elevated risk within the cylinder as compared with outside the cylinder. The observed test statistic is obtained by calculating the likelihood ratio maximized over the collection of zones in the alternative hypothesis. The p value for the detection of clusters is calculated by using the Monte Carlo hypothesis testing (where a number of random replications of the dataset under the appropriate null hypothesis are generated, their test statistics computed and then compared with the observed test statistic to obtain the p-value). The null hypothesis is that the risk of malaria inside and outside the scanning window is the same.”**

Q3) The proportion of vectors biting outdoors was compared for the areas. This would mean ignoring the absolute biting frequency which differs largely between areas.

**A3: We have included the absolute biting frequencies for each area as shown in supplementary Table 2 below:**

**Result section**

**“There were more Anopheles mosquitoes collected outdoors in all geographical areas except area 6, where most of the mosquitoes were collected indoor (Table 2). The frequencies of vectors collected in each geographical area are summarized in Supplementary Table 2.”**

**References**

1. Royston P, Sauerbrei W: **Building multivariable regression models with continuous covariates in clinical epidemiology--with an emphasis on fractional polynomials.** Methods Inf Med 2005, **44:**561-571.
2. Sauerbrei W, Meier-Hirmer C, Benner A, Royston P: **Multivariable regression model building by using fractional polynomials: description of SAS, STATA and R programs.** Computational Statistics & Data Analysis 2006, **50:**3464-3485.
3. Kezdi G: **Robust Standard Error Estimation in Fixed-Effects Panel Models.** 2003.
